# Structural analysis of PLD3 reveals insights into the mechanism of lysosomal 5′ exonuclease-mediated nucleic acid degradation

**DOI:** 10.1093/nar/gkad1114

**Published:** 2023-11-22

**Authors:** Yvette Roske, Cedric Cappel, Nils Cremer, Patrick Hoffmann, Tomas Koudelka, Andreas Tholey, Udo Heinemann, Oliver Daumke, Markus Damme

**Affiliations:** Structural Biology, Max-Delbrück-Center for Molecular Medicine in the Helmholtz Association (MDC), 13125 Berlin, Germany; Biochemical Institute, Kiel University, Kiel, Germany; Leibniz-Institut für Molekulare Pharmakologie (FMP), Robert-Rössle-Straβe 10, 13125 Berlin, Germany; Biochemical Institute, Kiel University, Kiel, Germany; Institute of Experimental Medicine, Kiel University, 24188 Kiel, Germany; Institute of Experimental Medicine, Kiel University, 24188 Kiel, Germany; Structural Biology, Max-Delbrück-Center for Molecular Medicine in the Helmholtz Association (MDC), 13125 Berlin, Germany; Institute for Chemistry and Biochemistry, Freie Universität Berlin, 14195 Berlin, Germany; Structural Biology, Max-Delbrück-Center for Molecular Medicine in the Helmholtz Association (MDC), 13125 Berlin, Germany; Institute for Chemistry and Biochemistry, Freie Universität Berlin, 14195 Berlin, Germany; Biochemical Institute, Kiel University, Kiel, Germany

## Abstract

The phospholipase D (PLD) family is comprised of enzymes bearing phospholipase activity towards lipids or endo- and exonuclease activity towards nucleic acids. PLD3 is synthesized as a type II transmembrane protein and proteolytically cleaved in lysosomes, yielding a soluble active form. The deficiency of PLD3 leads to the slowed degradation of nucleic acids in lysosomes and chronic activation of nucleic acid-specific intracellular toll-like receptors. While the mechanism of PLD phospholipase activity has been extensively characterized, not much is known about how PLDs bind and hydrolyze nucleic acids. Here, we determined the high-resolution crystal structure of the luminal N-glycosylated domain of human PLD3 in its apo- and single-stranded DNA-bound forms. PLD3 has a typical phospholipase fold and forms homodimers with two independent catalytic centers via a newly identified dimerization interface. The structure of PLD3 in complex with an ssDNA-derived thymidine product in the catalytic center provides insights into the substrate binding mode of nucleic acids in the PLD family. Our structural data suggest a mechanism for substrate binding and nuclease activity in the PLD family and provide the structural basis to design immunomodulatory drugs targeting PLD3.

## Graphical Abstract

**Graphical Abstract:**
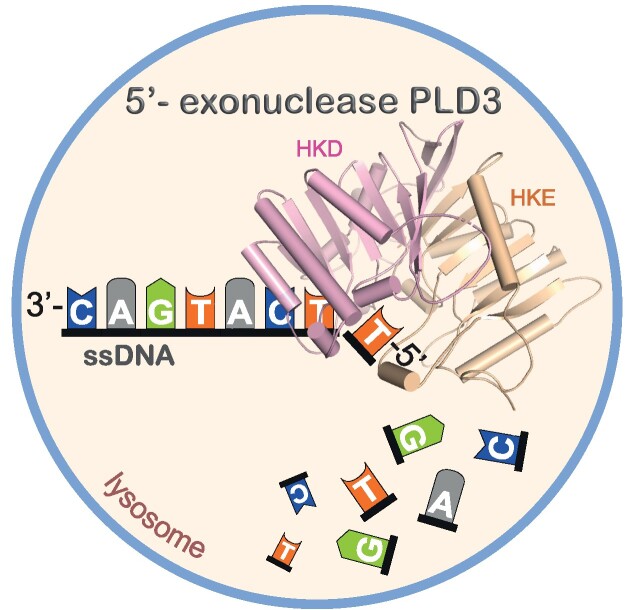

## Introduction

The intracellular lysosomal catabolism of complex macromolecules like proteins, (glyco-) lipids, glycosaminoglycans, N- and O-glycans, or nucleic acids by lysosomal enzymes is a highly coordinated process, mediated by the concerted action of >60 enzymes and additional accessory proteins. Amongst the poorly defined degradative pathways is the complete hydrolysis of nucleic acids: Both intracellular and extracellular RNA and DNA reach lysosomes by autophagy (e.g. cytosolic mRNA, tRNA, ribosomal RNA, mitochondrial RNA/DNA or nuclear DNA by piecemeal-autophagy of the nucleus) and, in a subset of cell types, by phagocytosis of foreign pathogens (bacteria, viruses) or self debris (e.g. due to the phagocytosis of apoptotic cells) (1). The amount of nucleic acids that is turned over in lysosomes is remarkable: it was found that in rat liver under conditions of nutritional deprivation, 65% of total cytoplasmic RNA is degraded per day, with approximately 70–85% occurring in lysosomes (2), highlighting the need for effective degradation. Notably, the efficient turnover of nucleic acids has an additional important physiological function: Late endosomes and lysosomes serve as critical organelles in the sensing of a subpopulation of intracellular nucleic acid innate immune system receptors, namely intracellular toll-like receptors (TLRs) (3). Intracellular TLRs bind both DNA and RNA as specific ligands. Upon dimerization, they transmit their signal to intracellular signaling cascades, finally activating NFκB and, consequently, pro-inflammatory gene expression and interferon regulatory factors (IRFs), inducing an interferon response. Thus, TLR-mediated signaling renders the endo-lysosomal turnover of nucleic acids in a well-adjusted and well-controlled manner critical for a balanced immune response against pathogens and autoimmunity (3–5). Therefore, a complete perception of the lysosomal degradation of nucleic acids is critical for understanding endosomal innate immunity and poses pharmacological intervention as an interesting approach to manipulating these processes.

After reaching the acidic compartments, the degradation of DNA to nucleotides is initiated by the cleavage of DNase II, an endonuclease that cleaves double-stranded DNA with low sequence specificity (6). DNase II is ubiquitously expressed, and its deficiency in mice leads to the accumulation of apoptotic nuclei in phagocytic cells (e.g. macrophages) (7), highlighting its critical function in the initiation of the turnover of double-stranded DNA (dsDNA). To which extent (i.e. length of DNA fragments) hydrolysis occurs remains to be elucidated. However, it is assumed that DNase II generates smaller single-stranded DNA (ssDNA) fragments that are subject to further ssDNA-specific exonuclease activity. Indeed, an acidic ssDNA-specific 5′ DNase was already biochemically characterized in 1968 and assigned as ‘spleen exonuclease’ (8), but the coding gene was not cloned, and the protein was not identified. In 2018, ‘spleen exonuclease’ was identified as phospholipase D3 (PLD3) (5). The lysosomal degradation of RNA is mainly mediated by RNaseT2, which is an endonuclease that cleaves single-stranded RNA into mono- or oligonucleotides with generally little sequence specificity (9). In fact, PLD3 cleaves ssRNA with similar high efficiency to ssDNA (4,10). PLD3 is a resident lysosomal protein and is synthesized as an N-glycosylated type II transmembrane protein, which undergoes proteolytic processing in acidic compartments, yielding a soluble, stable luminal C-terminal domain (11). The type II topology determines the C-terminal domain, containing the active site, to be in the lumen of the lysosome. The transport of PLD3 involves the ubiquitination of the cytosolic N-terminus and subsequent sorting into intraluminal vesicles, where proteolytic processing occurs (11). Genetic variants in *PLD3* have previously been shown to double the risk of developing Alzheimer's disease (12). This genetic link, however, could not be reproduced by others and is still controversial (13–17). PLD3 has a paralog, PLD4, that has a similar 5′-exonuclease activity (5). In contrast to PLD3, which is broadly expressed in cell lines and tissues, the expression of PLD4 is restricted to myeloid cells. Notably, variants in *PLD4* are genetic risk factors for lupus erythematodes and autoimmunity (18). *In vivo*, PLD3 and PLD4 have (at least partially) redundant functions: the knockout of both in mice leads to death in the first weeks of life (5). Both proteins play a relevant role in the innate immune system: In myeloid cells from *Pld3* or *Pld4* knockout mice, the accumulation of short ssDNA or ssRNA fragments continuously triggers the endo-lysosomal TLRs TLR9 and TLR7, respectively, leading to an autoimmune response and inflammation mediated by NF-κB (4,5). The precise source of nucleic acid(s) (self vs. foreign, taken up by phagocytosis vs. autophagy) that drives TLR activation in PLD3/PLD4 deficient cells, however, remains enigmatic. More recently, mitochondrial DNA was identified as a major endogenous physiological substrate of PLD3, which, upon leakage from lysosomes deficient in PLD3, can activate cytoplasmic STING (19).

While crystal structures for RNaseT2 and DNase II are available and provide important insight into the molecular details and function of both enzymes (9,20), no experimentally determined structural information is available for the 5′ exonucleases PLD3 or PLD4. Both PLD3 and PLD4 belong to the phospholipase-D (PLD) family of phosphodiesterases, a protein family that shares a common biochemical mechanism and an H(X)K(X4)D (HKD) signature motif within their active site (21). HKD motifs represent linear sequences containing the amino acid residues histidine, lysine, and aspartate and are present in other PLDs, even though the second HKD motif found in the PLD3 sequence contains glutamate instead of aspartate (HKE) (22). The PLD family consists of six members in mammals: PLD1–PLD6. The most prominent family members of the PLD family are PLD1 and PLD2, cytosolic enzymes catalyzing the hydrolysis of phosphodiester bonds within phospholipids such as phosphatidylcholine and producing phosphatidic acid and choline as catabolic end products, thereby acting as critical mediators of intracellular signaling pathways. Little is known about PLD5, and it is even unclear if it is active as an enzyme. PLD6 (synonymously called MitoPLD due to its localization in mitochondria) and its Drosophila homolog *zucchini* share some similar features with PLD3 and PLD4: It also contains a transmembrane segment and is, interestingly, active as a nuclease. PLD6 bears endoribonuclease activity for single-stranded RNAs *in vitro*, and the RNA cleavage products bear a 5′-monophosphate group (23,24). In contrast to PLD3 and PLD4, which contain two HKD/HKE motifs, the catalytic center of PLD6/*zucchini* is formed by the intermolecular homodimerization of two polypeptides containing one HKD motif each. Notably, this kind of homodimerization is quite common among the PLD family members that act as nucleases (21). Besides the six PLD-family members in humans and many eukaryotes, a plethora of family members have been identified and functionally characterized in viruses, prokaryotes, and plants, having both phosphodiesterase activity towards lipids as phospholipase D enzymes as well as hydrolytic activity towards nuclei acid as nucleases. *Nuc* is an endonuclease found in *Escherichia coli*, and its structure determination yielded important initial insight into the catalytic mechanisms of PLD-family member nucleases (25). The histidine from one of the two HKD motifs was suggested to act as a nucleophile in the catalytic mechanism, forming a phosphoenzyme intermediate, whereas a histidine residue from the other motif appears to function as a general acid in the cleavage of the phosphodiester bond. The lysine residues are involved in phosphate binding (25).

Despite the structural and biochemical characterization of *Nuc*, remarkably, no structural information of a PLD family member with a bound DNA/RNA has been reported. Consequently, the substrate binding mode and structural details of the catalysis are not available. Here, we show the crystal structure of the luminal domain of PLD3 as an apoenzyme and in complex with its natural substrate, ssDNA. Our findings help us understand the molecular mechanisms of the lysosomal PLD3-mediated single-stranded nucleic acid cleavage and might be helpful for designing immunomodulatory drugs targeting PLD3.

## Materials and methods

### Cell lines, antibodies and reagents

HEK293S GnTI^-^cells were obtained from Dr Norbert Sträter, Leipzig, Germany. HeLa cells were purchased from CLS Cell Lines Service GmbH (Germany). An antibody against the luminal domain of PLD3 (HPA012800) was purchased from Sigma-Aldrich. The antibody against LAMP2 (clone H4B4) was purchased from the Developmental Studies Hybridoma Bank, Iowa City. The antibody against the KDEL epitope (clone 10C3) was purchased from Millipore. Chemicals and reagents were purchased, if not otherwise indicated, from Sigma-Aldrich.

Unmodified and phosphorothioate-modified oligodeoxynucleotides (oligos) were custom synthesized by Biomers and purchased as mixed chiral (Rp)- and (Sp)-configured enantiomers.

### hPLD3 expression system

For large-scale recombinant production and purification, the DNA sequence of the luminal domain (residues 61–490) of human PLD3 (from here hPLD3) was fused to that encoding a C-terminal 6× histidine (6× His) tag and an N-terminal Igκ-signal sequence in the pcDNA3.1/TnhEF-SB9-cl1-real vector (gift from Dr Manfred Gossen), in which the expression of the gene product is controlled by a eukaryotic elongation factor 1 subunit α (EF-1α) promoter and flanked by two transposons for the sleeping beauty transposase (26). Co-transfection with the SB100-transposase containing vector (pCMV(CAT)T7-SB100(AL) SB100-Transposase; a gift from Dr Manfred Gossen) leads to integration of the hPLD3 sequence together with a puromycin resistance gene into the nuclear genome of the host cell line. As a host cell line, HEK293S GnTI^-^ was chosen: This cell line lacks *N*-acetylglucosaminyltransferase I (GnTI) activity and is often used for the production of recombinant proteins carrying homogenous high mannose-type glycan structures (27). After the generation of monoclonal producer cell lines, the selected cell lines expressed and secreted hPLD3 into their culture supernatant.

### Purification of hPLD3 for crystallography

The stably transfected HEK293S GnTI^-^ monoclonal cell line was raised in T175 cell culture flasks (Sarstedt) in growth medium (DMEM containing 10% (v/v) fetal calf serum) containing 7 μg/ml puromycin (InvivoGen) to ∼90% confluency, followed by the addition of production medium (DMEM containing 2.5% (v/v) fetal calf serum) for seven days. For harvesting the conditioned media, cells and cell debris were removed by centrifuging for 5 min at 500 g, followed by sterile filtration. The cell culture supernatant was concentrated to a final volume of 50 ml and purified on an AEKTA Purifier system using the HisTrap™ HP Ni-charged IMAC column (GE Healthcare). After NiNTA affinity purification, protein fractions were eluted with PBS containing 250 mM imidazole, pooled and further purified by size exclusion chromatography (SEC) on the HiLoad™ 16/600 Superdex™ 75 prep grade column (GE Healthcare) in HEPES buffer at pH 7.5 containing 150 mM NaCl. The protein was concentrated in a Vivaspin® 20 ultrafiltration unit (cutoff 10 kDa) at 4300 g and 4°C to a final concentration of 10–20 mg/ml, followed by snap freezing in liquid nitrogen. The protein concentration was measured by UV-spectroscopy from 240 to 320 nm in the Tecan SPARK® photometer. Purity was confirmed by SDS-PAGE and colloidal Coomassie stain.

For the determination of the purified protein's molecular weight, size exclusion chromatography was performed on a HiLoad 16/600 Superdex 200 pg column (Cytiva) in HEPES buffer (pH 7.5). The column was calibrated with a mixture of protein standards from the gel filtration LMW and HMW calibration kits (Cytiva) with the same running parameters as for the recombinant protein.

### Lysosome enrichment from the mouse liver and digestion of hPLD3

The enrichment of lysosomes from the mouse liver was performed as described previously (28). 20 μg of hPLD3 were incubated without or with 5 μg of lysosome-enriched fractions for 3 h and for 18 h in 50 mM sodium acetate buffer containing 100 mM NaCl and 5 mM l-cysteine at pH 4.5. The reaction was stopped by the addition of Lämmli buffer and heating to 95°C, followed by SDS-PAGE and Coomassie staining.

### PLD3 enzyme activity assay

The specific enzymatic activity of PLD3 was determined at the optimal acidic pH of 6.0 as described previously by the End-labeled Fluorescence-Quenched Oligonucleotide (EFQO) assay based on the measurement of fluorescence emission upon the release of a quencher from a fluorophore-labeled oligonucleotide after enzymatic digestion (29).

### Crystallization and structure determination

Apo-hPLD3 in 20 mM HEPES pH 7.0, 150 mM NaCl was crystallized using the sitting-drop vapor-diffusion method. Crystallization setups were performed by using a Gryphon pipetting robot (Matrix Technologies Co.) for pipetting 200 nl of protein with a concentration of 10 mg/ml to an equal volume of precipitant solution. The Rock Imager 1000 storage system (Formulatrix) was used for storing and imaging the experiments. Crystals appeared within 3 days. For crystallographic phase determination, the crystals grew in 17% PEG 4000, 0.02 M NaI, 0.6 M ammonium sulfate, 15% glycerol was soaked in 0.5 mM ammoniumtetrachloroplatinate overnight before flash-freezing in liquid nitrogen. A 1.99 Å data set with an anomalous signal up to 2.99 Å was collected at a wavelength of λ=1.072Å. A second native 1.51 Å apo-data set was measured at λ=0.98141 Å from a crystal grown under identical conditions but containing 0.75 M ammonium sulfate. At the identical wavelength, a 1.85 Å dataset of hPLD3 co-crystallized and additionally soaked for 2 days with the phosphorothioate-modified ssDNA oligo T*TCATG was collected. Crystals were grown in 15% PEG 4000, 0.02 M NaI, 0.9 M ammonium sulfate, 15% glycerol and were directly flash-frozen for data collection.

All diffraction data were recorded on BL14.1 at BESSY II (Helmholtz-Zentrum Berlin, HZB), processed, and scaled using XDSapp (30). The crystallographic phase problem for the ammoniumtetrachloroplatinate derivative of hPLD3 was solved by using HKL2Map (31), and the initial model was built by ARP/wARP (32). The structure of oligo-bound hPLD3 was solved by molecular replacement with Phaser (33) using the native apo-hPLD3 structure as the search model. Finally, both hPLD3 structures were manually built using COOT (34) and iteratively refined using Refmac (35) and Phenix (36).

Amino acids 72–98 and 101–490 of the hPLD3 structures are well explained in the electron density, whereas residues 61–71 and 99–100 were not visible in the electron density. 97.5% of the residues in the apo hPLD3 structure are in the allowed regions of the Ramachandran plot, and 97.1% for the hPLD3-T*TCATG oligo complex structure. The Ramachandran statistics of both structures were analyzed using Molprobity (37). Figures and structure superimpositions were generated with PyMol (http://www.pymol.org). Protein interfaces were calculated by the Protein Interfaces, Surfaces, and Assemblies (PISA) server (38).

The atomic coordinates of apo hPLD3 and hPLD3-T*TCATG complex have been deposited in the Protein Data Bank (PDB ID codes 8Q1K and 8Q1X, respectively).

### Analytical ultracentrifugation

Sedimentation velocity (SV-AUC) experiments of hPLD3 were carried out at 40000 rpm, at 20°C. Sample solutions were analyzed at a final concentration of 8 or 16 μM hPLD3. Absorbance data were acquired at a wavelength of 280 or 290 nm and in time intervals of 5 min. Sedimentation coefficient distributions c(s) were analyzed with the program Sedfit (39). The protein partial-specific volume and the buffer physical constants were calculated from amino acid and buffer composition, respectively, using SEDNTERP (40).

### Isothermal titration calorimetry (ITC)

ITC experiments were performed using a PEAQ-ITC microcalorimeter (Malvern). All titrations were performed at 10°C with 380 μM ssDNA variants (TTCATG, T*TCATG, T*T*C*A*T*G*, T*T) in the syringe and 10 μM hPLD3 in the reaction chamber. We also doubled the hPLD3 concentration in the reaction chamber to 20 μM and obtained comparable values, but with higher errors due to the unfavourable protein:DNA concentration ratio owing to the limitation of the ssDNA stock concentration of 400 μM. The protein as well as the titration components were in buffer containing 20 mM HEPES pH 7.5, 150 mM NaCl. Malvern software was used for data fitting. At the applied protein concentration of 10 and 20 μM, hPLD3 is mostly dimeric (see AUC data in Figure 3D).

### Mass spectrometry

#### Sample processing for analysis of hPLD3 after incubation with mouse liver lysosomes

After incubation of hPLD3 with mouse liver lysosomes followed by SDS-PAGE and Coomassie staining, gel bands (0 h and 18 h) were excised from the gel and split into two halves to be processed by the two proteases pepsin and elastase, respectively. The halves were further cut into 2 mm^3^ cubes and destained with 100 mM ammonium bicarbonate (ABC), 30% acetonitrile (ACN), 50 mM ABC and 100% ACN. Samples were reduced with dithiothreitol (10 mM) at 65°C for 30 min and then alkylated with iodoacetamide (55 mM final) in the dark at room temperature for 30 min. Gel bands were dehydrated using ACN, and then the buffer was exchanged using HEPES buffer (100 mM pH 7.0) and dehydrated again. Reductive dimethylation of free amino groups was performed using 30 mM formaldehyde and 15 mM sodium cyanoborohydride (200 mM HEPES, pH 7) for 8 h at 37°C. The reaction was quenched by adding 0.9 M Tris buffer (overnight). Gel pieces were then washed, dehydrated, and the protein deglycosylated by the addition of PNGaseF (100 units) overnight in the presence of ABC buffer at 37°C. Gel pieces were dehydrated and then incubated (overnight, 37°C) with 200 ng of either pepsin in the presence of 10 mM HCl or elastase in the presence of ABC buffer. Eluted peptides were then subjected to LC–MS analysis.

#### LC–MS analysis of hPLD3 after incubation with mouse liver lysosomes

Protein digests were injected on a Dionex Ultimate 3000 nano-UHPLC coupled to a Q Exactive mass spectrometer (Thermo Scientific, Bremen). The samples were washed on a trap column (Acclaim Pepmap 100 C-18, 5 mm × 300 μm, 5 μm, 100 Å, Dionex) for 2 min with 3% ACN/0.1% TFA at a flow rate of 30 μl/min prior to peptide separation using an Acclaim PepMap 100 C18 analytical column (50 cm × 75 μm, 2 μm, 100 Å, Dionex). A flow rate of 300 nL/min using eluent A (0.05% formic acid (FA)) and eluent B (80% ACN/0.04% FA) was used for gradient separation (5–40% B). Spray voltage was applied on a metal-coated PicoTip emitter (10 μm tip size, New Objective, Woburn, Massachusetts, US) with a source temperature of 250°C. Full scan MS spectra were acquired between 300 and 1500 *m*/*z* at a resolution of 70 000 at *m*/*z* 200, and the top 10 most intense precursors with charge state greater than 2+ were selected for fragmentation using an isolation window of 1.6 m/z and with HCD normalized collision energies of 27 at a resolution of 17 500. Lock mass (445.120025) and dynamic exclusion (20 s) were enabled.

#### LC–MS of hPLD3 used for crystallization

Mass spectrometry analysis was performed using liquid chromatography–electrospray ionization-quadrupole-time of flight-mass spectrometry (LC–ESI-Q-TOF-MS) at the protein production and characterization platform at the Max-Delbrück-Centrum (MDC). Intact mass analysis was conducted on an Agilent 1290 Infinity II UHPLC system coupled to an Agilent 6230B time-of-flight (TOF) LC/MS instrument equipped with an AJS (Agilent Jet Stream technology) ion source operated in positive ion mode (denaturing conditions). 0.3 μg protein samples diluted in 0.1% FA were injected for each analysis. Mass spectrometry data were analyzed using the protein deconvolution feature of the MassHunter BioConfirm Version 10.0 software (Agilent). Deconvolution was performed between a mass range of 2000–70 000 m/z (mass-to-charge ratio).

#### Database search and statistics

The MS raw files were processed by Proteome Discoverer 2.2, and MS/MS spectra were searched using the Sequest HT algorithm against a database containing common contaminants, PNGaseF, recombinant PLD3 and the mouse database. The enzyme specificity was set to unspecific. An MS1 tolerance of 15 ppm and an MS2 tolerance of 0.02 Da was implemented. Oxidation (15.995 Da) of methionine residues, deamidated asparagine (0.0984 Da), dimethylation (28.031 Da), and glutamine to pyroglutamate (–17.027 Da) at the N-terminus were set as a variable modification. Carbamidomethylation (57.02146 Da) on cysteine residues was set as a static modification. Dimethylation on lysine residues was set either as a fixed modification on lysine residues or as a variable modification. The minimal peptide length was set to 7 amino acids, and the peptide false discovery rate (FDR) was set to 1%. In addition to the above-mentioned variable modifications, the spectra were also searched for cysteine oxidation, cysteine sulfonate, i.e. trioxidation (47.9847 Da) was set as a variable modification along with carbamidomethylation.

#### Transfection of HeLa cells

Plasmid-DNA was delivered into eukaryotic cells by transfection using polyethylenimine (PEI) (Sigma Aldrich): 1 μg of plasmid DNA was mixed with 7.5 μl PEI and 300 μl of DMEM (Gibco), incubated for 20 min at room temperature, and added to the fresh growth medium (3 ml on a 10 cm dish). The transfection was performed at 37°C with 5% CO_2_ for 5 h with the subsequent addition of fresh growth medium containing 10% fetal calf serum.

#### Indirect immunofluorescence

Cells were seeded on sterile glass coverslips in a 6-well plate (four coverslips per well) or in a 24-well plate (one coverslip per well). After cultivation and transfection where necessary, the cells were washed twice with cold PBS and fixed with ice-cold methanol for 20 min followed by two additional washing steps in cold PBS. For immunofluorescence staining, the cells were permeabilized in 0.2% saponin in PBS for 5 min, followed by quenching in 0.12% glycine in 0.2% saponin/PBS for 10 min. After washing in 0.2% saponin/PBS once, the cells were blocked in 10% FCS in 0.2% saponin/PBS for 30–60 min. Primary antibodies were diluted in 10% FCS/0.2% saponin/PBS and incubated overnight at 4°C. The next day, the coverslips were washed four times in 0.2% saponin/PBS and incubated in a 1:500 dilution of AlexaFluor-conjugated secondary antibodies (ThermoScientific) for 1 h at room temperature, followed by another four washing steps in 0.2% saponin/PBS and two washing steps in H_2_O. Finally, the cells were mounted with 15 μl mounting medium (167 mg/ml Mowiol® 4–88, 3% glycerol, 20 mg/ml 1,4-diazabicyclooctane (DABCO®) in PBS) containing 1 μg/ml DAPI and dried at 4°C.

### Statistical analysis

All statistical analyses were performed using GraphPad Prism version 7.04 or later. Curves, bar graphs, and scatter plots show mean values with error bars indicating the calculated standard deviation wherever available. For the comparison of groups with a single control group, a one-way ANOVA was chosen for significance analysis using Dunett's test, correcting for multiple comparisons to a significance level of α ≤ 0.05.

For multiple comparisons of groups with each other, a one-way ANOVA was chosen for significance analysis using Tukey's range test correcting for multiple comparisons to a significance level of α ≤ 0.05. For the comparison of only two groups, a two-sided *t*-test was performed on a significance level of α ≤ 0.05.

## Results

### The PLD3 luminal domain has an N-terminal pyroglutamate

**Figure 1.**
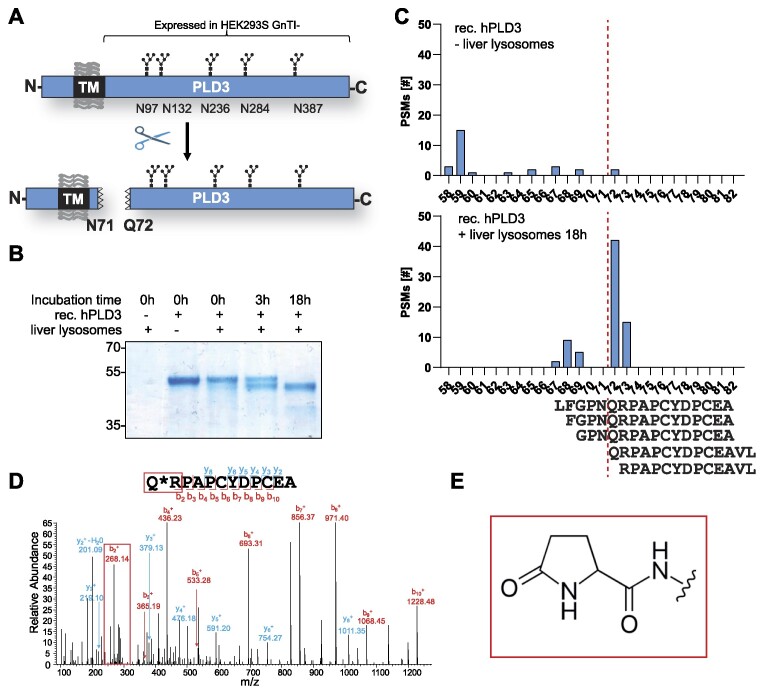
PLD3 is converted from its transmembrane form into a soluble form, and the neo-N-terminus is Q72. (**A**) Schematic drawing of the topology and proteolytic conversion of PLD3 from its transmembrane form to the soluble C-terminal domain. Putative N-glycosylation sites are depicted. The C-terminal domain (amino acids 61–490) was expressed in HEK293S GnTI^-^ cells. (**B**) Coomassie-stained SDS-PAGE gel of recombinant hPLD3 (amino acids 61–490) after treatment with purified mouse liver lysosomes (5 μg) for 0, 3 and 18 h. Purified mouse liver lysosomes without rec. hPLD3 is depicted in the left lane. (**C**) Number of peptide spectrum matches (PSMs) for neo-N-terminal peptides detected by MS/MS at 0 h of digestion and after 18 h of digestion with purified liver lysosomes. The peptide sequence of the most abundant peptides is shown (**D**) MS/MS spectrum of the most abundant neo-N-terminal peptide after 18 h of digest Q*RPAPCYDPCEA. The b-series ion of the pyroglutamate (128.1307–17.0305) +Arginine (156.1011) + H (ion on the N-terminus) is boxed in red. (**E**) Structure of the N-terminal pyroglutamate with the subsequent peptide bond.

PLD3 is synthesized as a transmembrane protein with a type II topology. Upon reaching acidic compartments, it is cleaved by an unknown protease releasing the soluble luminal domain (Figure 1A) (11). In order to elucidate the structure of hPLD3 and to determine the exact cleavage site at the neo-N-terminus, we recombinantly expressed the luminal domain of human PLD3 (residues 61–490, from here on hPLD3) starting directly after the transmembrane segment (Supplementary Figure S1A-C). hPLD3 was expressed in HEK293S GnTi^-^ cells producing a homogenous N-glycosylation pattern (27) and purified from the cell culture supernatant by NiNTA affinity chromatography. Digestion of the recombinant protein with PNgase F or EndoH confirmed the high-mannose-type N-glycosylation (Supplementary Figure S1D). To determine its neo-N-terminus, we incubated the purified recombinant protein with catalytic amounts of purified lysosomal fractions, followed by mass spectrometry to identify the neo-N-terminus. Upon extended digestion, a clear shift in the molecular weight of the luminal domain of PLD3 was discernable (Figure 1B). While the C-terminus was still intact, we observed considerable trimming of the amino-terminus, presumably due to exopeptidase activity from the liver lysosomes. Mass spectrometry analysis of the recombinant protein identified a number of prominent peptides starting with Gln72, suggesting that this amino acid is the neo-N-terminus after proteolytic digestion (Figure 1C). Interestingly, this glutamine residue was modified to a pyroglutamate, a derivative in which the free amino group of glutamic acid or glutamine spontaneously cyclizes to form a lactam (Figure 1D, E) (41). These data indicate that the luminal domain is likely N-terminally proteolytically processed up to the pyroglutamate 72, which seems to form a stable neo-N-terminus.

### The structure of hPLD3

To gain insight into hPLD3 architecture, we determined the crystal structure of hPLD3 in the substrate-free (apo) form to a resolution of 1.5 Å (Supplementary Table S1). The phase problem was solved by a single anomalous scattering protocol. The asymmetric unit contained two PLD3 molecules. In both molecules, amino acids 72–98 and 101–490 were well explained in the electron density, whereas residues 61–71 and 99–100 were not visible, likely due to their exposure to the solvent providing them with higher flexibility. The model was refined to *R*_work_/*R*_free_ of 15.4%/18.6%.

**Figure 2.**
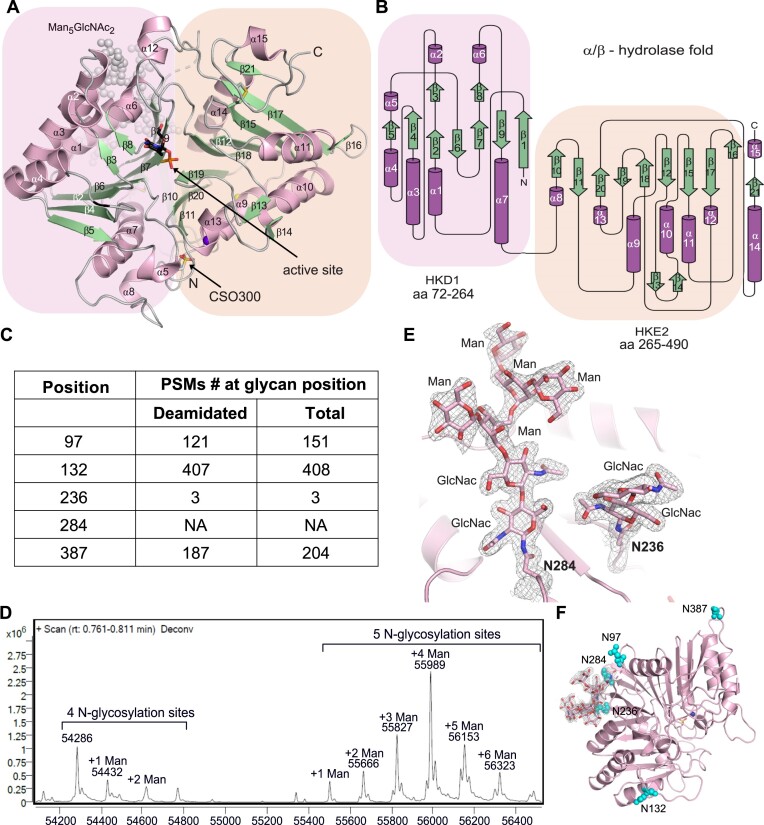
The N-glycosylated PLD3 structure. (**A**) The detailed structure of one protomer (chain B) of the PLD3 homodimer is shown as a cartoon representation with β-strands depicted in green and α-helices in pink in complex with the ssDNA oligo thymidine product presented as black sticks in the active site. The N-glycosylation with Man_5_GlcNAc_2_ is highlighted as gray spheres in the background. The HKD1 motif is boxed in pink, and the HKE2 motif is in wheat color. Cysteine 300 hyperoxidized to cysteine sulfonic acid (OCS) is depicted as a stick model. (**B**) Structural topology diagram of a PLD3 protomer with the same color code for the β-strands and α-helices as well as the HKD/HKE motif as in (A). (**C**) Table with the number of deamidated peptide spectrum matches (PSMs) and the total number of PSMs are depicted, where N-glycosylation can be assumed (from Uniprot and Glygen.org/ Q8IV08/) as assessed by mass spectrometric analysis of the recombinant hPLD3 after tryptic digestion. (**D**) LC–MS mass spectrometry analysis of hPLD3 (61-490) showing the number of N-glycosylated residues with Man_5_GlcNAc_2_ plus their extension with different numbers of mannose sugar moieties. (**E**) Magnified view of the two observed N**-**glycosylated amino acids N284 and N236 in hPLD3 are presented here with the covalently linked sugar molecules in stick presentation, surrounded by the 2Fo-Fc electron density contoured at 1 sigma. Electron density for only two GlcNac sugar moieties is observed for N236, whereas N284 contains a linked Man_5_GlcNAc_2_. The N-glycosylation is shown here in the protomer A from the hPLD3 apo structure. The same N-glycosylation pattern is found for protomer B and in the oligo-bound hPLD3. (**F**) All N-glycosylation sites surrounding the hPLD3 are shown as cyan spheres.

Similar to other HKD family members, hPLD3 has an α/β-hydrolase fold consisting of a central β-sheet core sandwiched between two α-helical layers (Figure 2A, B). The active site with the signature H(X)K(X4)D (HKD) sequences is formed by residues located in the HKD1 (aa72-264) and HKE2 (aa 265–490) domains. The architecture is related to the reported structures of other PLD superfamily members, such as *Nuc*, PLD6, and phospholipase D, and the predicted structure of PLD4 (Supplementary Figure S2A-D).

PLD3 contains five predicted N-glycosylation sites. We confirmed four of them by mass spectrometry after tryptic digestion (Figure 2C). However, LC-MS of the non-digested protein under denaturing conditions of hPLD3_61-490_ revealed several peaks in the mass range of 55 600–56 400 Da, which corresponds to hPLD3 N-glycosylated at five sites, each harboring a high-mannose-type sugar (plus Man_5_GlcNAc_2_ reveals a total mass of 55429.9 Da) plus 1–6 additionally linked mannose moieties (each 162 Da) (Figure 2D). In the lower mass range of 54 200–54 800 Da, several peaks are observed, which depict hPLD3 N-glycosylated only at four sites with further linked mannose residues ranging from one to three. Notably, an additional mass difference of about 80 Da was observed for all hPLD3 peaks in the spectra of the full-length protein, which may represent an additional sulfonate site.

In line with the mass spectrometry data, a high mannose-type sugar (Man_5_GlcNAc_2_) was well visible in the electron density at Asn284 (Figure 2E). For Asn236, electron density could be observed for the two N-acetylglucosamines (GlcNAc), whereas the remaining sugars are likely disordered (Figure 2E). For the remaining three N-glycosylation sites, the sugars are not visible, likely because they are located in flexible loop regions (Figure 2F). In summary, our data indicate that all five N-glycosylation sites (e.g. Asn97, Asn132, Asn236, Asn284 and Asn387) are at least partially N-glycosylated with Man_5_GlcNAc_2_ but only two of them are defined in the crystal structure. Notably, a mutation in one of the N-glycosylation sites, the N284S variant, was suggested to increase the risk of Alzheimer's disease (Supplementary Figure S2E).

The electron density also indicated that the side chain of Cys300 was oxidized to sulfonic acid. The cysteine sulfonic acid (OCS) residue is located in a deep pocket 20 Å away from the active site (Supplementary Figure S3A, B) and is coordinated by the side chain of Ser294, the carbonyl oxygen of Pro296 and water molecules. Since X-ray-induced photo-oxidation of cysteines in their most reactive ionized thiolate form (Cys-S^−^) has been observed occasionally (42), we validated the occurrence of the cysteine sulfonate in the recombinant protein before crystallization and X-ray exposure by mass spectrometry (Supplementary Figure S3C). In these experiments, Cys300 was the only cysteine found to be oxidized to a significant extent.

Since changes in the sulfur oxidation state of cysteines are often used to modify the catalytic activity of proteins (43,44) and since mutation of C300 was associated with the development of late-onset Alzheimer's disease (Supplementary Figure S2E), we investigated a possible effect on the hPLD3 5‘-exonuclease activity. However, the C300S mutant revealed no significant change in the nuclease activity compared to the wildtype (Supplementary Figure S3D). We furthermore tested if the mutagenesis of the cysteine to serine affects the localization or proteolytical processing of the full-length PLD3 in transfected cells. PLD3-C300S still localizes to lysosomes in transfected Hela cells and is still proteolytically processed (Supplementary Figure S3E, F).

In the same deep cavity of OCS300, we found electron density for a large cation that was coordinated to the oxidized cysteine side chain via a water molecule (Supplementary Figure S3B). Careful evaluation of the electron density in combination with an X-ray fluorescence scan of the crystal used for data collection of the hPLD3 apo structure indicated that this density corresponds to a manganese ion (Supplementary Figure S3G, H). In DNA hydrolysis assays, the addition of Mg^2+^ or Mn^2+^ to recombinant hPLD3 did not affect catalysis (Supplementary Figure S3I).

### hPLD3 forms a homodimer

**Figure 3.**
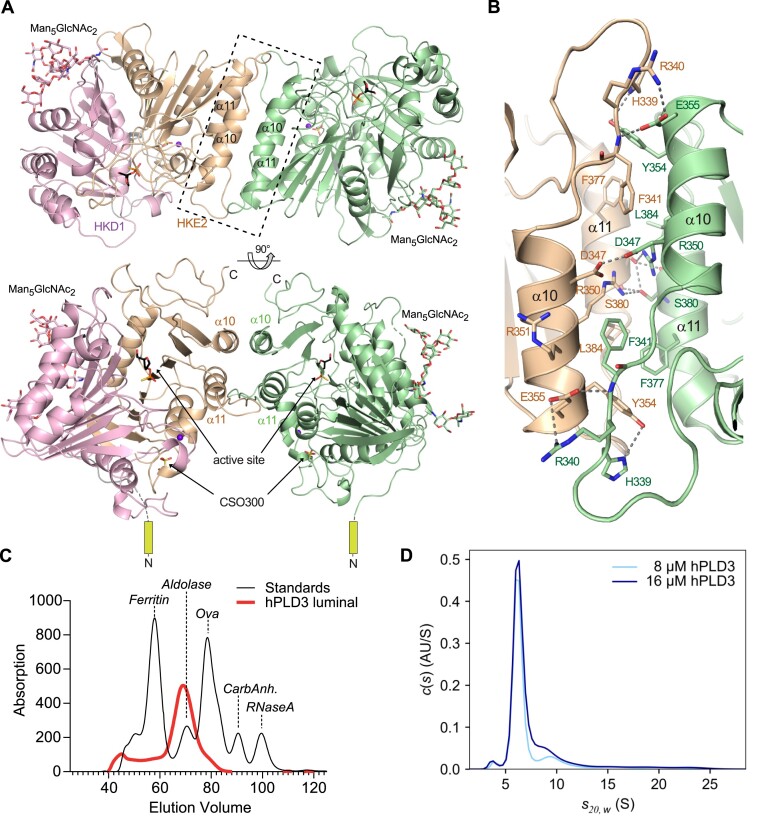
PLD3 forms a homodimer. (**A**) Orthogonal views of the hPLD3 homodimer bound to the ssDNA T*TCATG with one protomer shown in green and the other in pink and wheat color demonstrating the HKD1 and HKE2 motif (according to the color code in (A) and (B)) forming the active site harboring the ssDNA oligo cleavage product 3'-phosphorothioate-thymidine. The bound Man_5_GlcNAc_2_ molecules and the cysteine sulfonate (OCS300) are highlighted in stick presentation. The homodimer interface is depicted by a dashed box. (**B**) A magnified view into the homodimer interface formed by α-helices 10 and 11 shows the interacting residues. Hydrogen bonds are shown as dashed lines. (**C**) Superimposed elution profiles of low-scale preparative size exclusion chromatography using standard proteins (black) and the luminal hPLD3 (red). As standard proteins were used ferritin (400 kDa), aldolase (153 kDa), ovalbumin (44 kDa), carbonic anhydrase (29 kDa), ribonuclease A (14 kDa). (**D**) Sedimentation velocity (SV-AUC) experiments of hPLD3 at a final concentration of 8 (light blue) and 16 μM (dark blue) show a main peak at a sedimentation coefficient of about 6.3 S containing about 65 - 70 % of the loaded material. The estimated molecular weight of 96 kDa of this species is close to the size of a glycosylated hPLD3 homodimer (98 kDa). Two smaller peaks at 4.0 and 10.2 S correspond to populations of monomeric (3%) and tetrameric hPLD3 (10%), respectively.

The two hPLD3 molecules in the asymmetric unit formed a homodimer via helices α10 and α11 (Figure 3A, B), with an interface area of 1042 Å^2^. The dimer interface is formed by a central hydrogen bond network involving residues Asp347, Arg350 and Ser380, flanked by hydrogen bonds made by His339, Arg340, Tyr354, Glu355 and hydrophobic interactions between residues Phe377, Phe341 and Leu384 (Figure 3B). In agreement with a physiological function of the homodimer, the two N-termini pointed in the same direction so that the two N-terminal transmembrane helices of full-length PLD3 could insert together into one membrane surface. Notably, the PLD3 dimer has two complete catalytic sites, whereas the dimerization of other PLD family members containing only one HKD domain leads to a dimer with one catalytic site.

Analytical size exclusion chromatography (Figure 3C) and analytical ultracentrifugation experiments (Figure 3D) confirmed the presence of a hPLD3 dimer in solution. To test the functional relevance of dimer formation, double (R350A, S380A) and quadruple alanine mutations (R350A, S380A, Y354A, F377A) of residues involved in the dimer interface were generated and analyzed for their enzymatic activity. Both mutant versions were catalytically inactive upon transfection of the corresponding cDNAs in HeLa cells (Supplementary Figure S4A). Immunofluorescence analysis revealed the retention of the proteins in the endoplasmic reticulum, suggesting misfolding of the mutants (Supplementary Figure S4B). Accordingly, the double and quadruple hPLD3 mutant proteins were not properly proteolytically processed to the luminal domain (Supplementary Figure S4C). These data suggest a critical function of the dimerization interface for proper folding and stability.

### ssDNA oligomer binding in the active site

**Figure 4.**
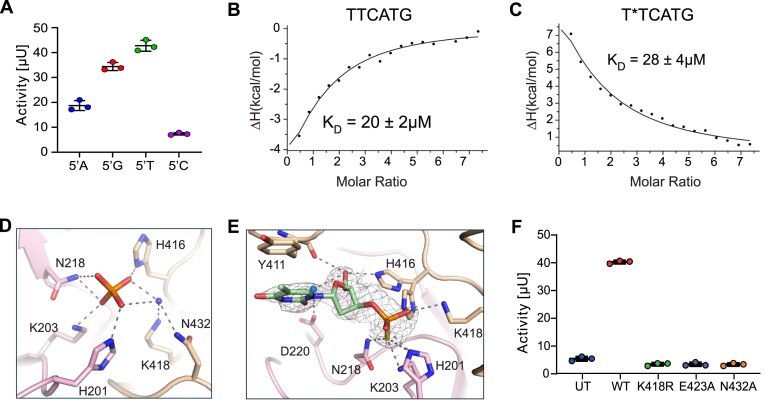
The active site of hPLD3. (**A**) hPLD3 activity assay with oligonucleotides harboring different 5′-nucleotides (5′-adenosine (5′A), 5′-guanosine (5′G), 5′-thymidine (5′T), 5′-cytidine (5′C)). (**B**) Isothermal titration calorimetry (ITC) data showing the binding of the ssDNA oligo TTCATG to hPLD3_61-490_ with a *K*_D_ of 20 ± 2 μM, Δ*H* = –12 ± 3 kcal/mol, –*T*Δ*S* = 5.7 kcal/mol, Δ*G* = –6.1 kcal/mol, *N* = 1.0 ± 0.10. (**C**) The phosphorothioate-modified ssDNA oligo T*TCATG binds with a *K*_D_ of 28 ± 4 μM, Δ*H* = 30 ± 5 kcal/mol, –*T*Δ*S* = –34.4 kcal/mol, Δ*G* = –5.9 kcal/mol, *N* = 0.9 ± 0.1 to hPLD in the ITC experiment. (**D**) The active site of the apo PLD3 structure is shown in the example of the protomer chain B with a bound phosphate coordinated by the residues of the HKD1/HKE2 motif or a water molecules (blue sphere). Hydrogen bonds are depicted as dashed lines. (**E**) The oligo T*TCATG binding in the active site is shown for the protomer molecule of chain B. Only electron density for the first thymidine of the T*TCATG oligo and the phosphorothioate was found in each of the two protomers of PLD3. Histidine 416 shows an alternative conformation, which indicates that it binds either to the phosphothioate or to the sugar base of the thymidine. Both conformations are shown. (**F**) Cell-based hPLD3 activity assay of lysates from cells transfected with hPLD3 mutated in residues K418R, E423A and N432A belonging to the HKD1/HKE2 motif. UT = untransfected cells; WT = cells transfected with wildtype PLD3.

We aimed to obtain further insight into hPLD3 substrate binding and catalytic activity. To this end, we tested single-stranded (ss) DNA oligomers with different 5′-ends as substrates for the 5′-exonuclease hydrolysis activity by hPLD3. We randomly mixed the remaining nucleotides so that they resembled a physiological oligonucleotide (Figure 4A). In agreement with previous results from cell-based assays (29), the highest activity was found for 5′ T-oligomers, followed by 5′ G-, 5′ A-, and low activity for 5′ C-oligomers.

To test the binding of 5′ T-oligomer to hPLD3, we designed four ssDNA oligos TTCATG, T*TCATG, T*T*C*A*T*G*, and T*T where a non-bridging oxygen in the phosphodiester bond is replaced by a sulfur atom (* phosphorothioate) to prevent 5′-exonuclease cleavage. Isothermal titration calorimetry (ITC) was used to investigate the binding of these phosphorothioate ssDNA oligos, which revealed only a binding for the unmodified TTCATG and the partly modified T*TCATG, with *K*_D_ of 20 and 28 μM, respectively (Figure 4B, C). The fully modified T*T*C*A*T*G* and the short T*T oligo did not bind to hPLD3 (Supplementary Figure S5A, B).

To obtain structural information, crystals of hPLD3 in the presence of the identified ss T*TCATG oligomer were grown and diffracted to 1.8 Å (Supplementary Table S1). The phase problem was solved by molecular replacement using the apo hPLD3 structure as a template. The structure was refined to a *R*_work_/*R*_free_ of 17.4%/19.9%.

The central active site of each protomer of hPLD3 in the apo and complex state is formed by the HKD/HKE-motif H_201_xK_203_(x)_4_D_208_(x)_6_GSxN_218_ and H_416_xK_418_(x)_4_E_423_(x)_6_GxSN_432_ (Figure 4D, E). His201, Lys203, and Asn218 from HKD1 as well as His416, Lys418, and Asn432 in the HKE2 motif, form the active site. Asp208 in HKD1 and Glu423 in HKE2 are located 20 Å distant from the active site and may contribute to the structural integrity of hPLD, as observed in phospholipase D family member PLD6 (24) and Nuc (25).

The hPLD3 apo structure harbors a bound phosphate in the active site, which is coordinated by five direct hydrogen bonds with the HKD/HKE motif residues His201, Lys203, Asn218, His416, and water-mediated contacts of Lys418 and Asn432 (Figure 4D).

The hPLD3 structure co-crystallized with the ssDNA phosphorothioate (*) modified T*TCATG oligomer revealed a partly visible DNA sequence bound within the central active site (Figure 4E). The 5′ end of the first thymidine and the phosphorothioate group of the second thymidine were bound in the active site. The phosphorothioate was coordinated by six hydrogen bonds within the HKD/HKE motif involving the active site residues His201, Lys203, Asn218, His416, Lys418, and Asn432 (Figure 4E). The thymidine was hydrophobically stacked against Tyr411 and was further stabilized by a hydrogen bond with Tyr126 (protomer A) or water-mediated contacts via Asp220 (protomer B) to the thymidine O2. The O5 of the deoxyribose was bound by the Tyr411 together with an alternative conformation of His416 (in protomer B) and water-mediated by Asp220 (protomer A) (Figure 4E). The remaining nucleotides of the T*TCATG oligo were not visible in the electron density. In line with our binding experiments, hPLD3 co-crystallization with combined soaking experiments with the unmodified ssDNA oligo variants TTCATG also revealed the same cleaved oligomer product in the active site.

Single mutations of active-site residues, K418A and N432A, resulted in a complete loss of the 5′-exonuclease activity compared to wildtype (Figure 4F), which confirms the contribution of these residues in forming a complete active site in hPLD3. The E423A mutation also showed a complete loss of 5′-exonuclease activity (Figure 4F). Notably, E423A was retained in the endoplasmic reticulum (ER) (Supplementary Figure S5C), whereas both K418R and N432A properly localized to lysosomes (Supplementary Figure S5C), supporting the critical role of Glu423 in structure stability and folding.

## Discussion

Upon arrival in acidic compartments, PLD3 is converted from its transmembrane form to the soluble form by unknown proteases. Under steady-state conditions, this soluble form is predominant in lysosomes, suggesting that this is the physiologically active form (11). We presumed that cleavage occurs between the transmembrane segment in the globular folded domain in an area with little secondary structure, and the results of our mass spectrometry analysis after digestion of the entire luminal domain with purified lysosomes confirmed this hypothesis. These results suggest that the C-terminal domain is gradually cleaved by aminopeptidases until a stable neo-N-terminus is reached, which was identified as pyroglutamate in position 72. Our approach, however, has some limitations. The *in vivo* proteolysis might be different due to the differential equipment of lysosomes with proteases in different cell types. Moreover, the exposure of the luminal domain to protease concentrations might be significantly different. However, the molecular weight of the *in vitro* trimmed luminal domain fits well with that generated *in vivo*, observed by immunoblot of tissues or cell lysates (11). Interestingly, the pyroglutamate might explain the high stability of this neo-N-terminus: The spontaneous cyclization of the N-terminus of amyloid β was shown previously to confer high resistance towards lysosomal cathepsin proteases (45). It is tempting to speculate that the spontaneous formation / cyclic epimerization of the N-terminus protects it from further exopeptidase-trimming under the harsh proteolytic conditions of the lysosome.

Crystal structures of hPLD3 in the apo and ssDNA oligomer-bound form revealed an overall closely related fold with other members in the PLD superfamily. DNA binding is not associated with major conformational changes (Supplementary Figure S2F), which is similar to phospholipid-binding in human PLD1 and PLD2 (46,47). An interesting finding from our study was that PLD3 forms homodimers. The dimerization interface is formed by the two helices α10 and α11 from each monomeric subunit. The hPLD3 protomers are oriented in such a way that the two N-termini of the hPLD3 monomers, where the transmembrane domains in full-length PLD3 are connected, point in the same direction (Figure 3A). This suggests that the full-length protein can also form such membrane-embedded dimers. Homodimerization is a common finding in the PLD family; however, in these cases, each protomer provides one HKD motif, and dimerization is, therefore, critical to forming the catalytic site. Notably, this homodimerization mode is especially typical for PLD members that act as DNA/RNA nucleases, while PLD enzymes active against lipids typically contain both active-site motifs in one polypeptide (21). In contrast to other PLD-related nucleases, hPLD3 and hPLD4 have two HKD/HKE motifs in one polypeptide that form the active site autonomously without the need for homodimerization. Instead, homodimerization in hPLD3 generates two catalytic sites that appear to be independent of each other. Mutagenesis of the hPLD3 dimerization interface leads to retention of the altered protein in the ER, presumably due to misfolding and ERAD-mediated degradation, preventing a more detailed analysis of the effect of homodimerization on its function and catalytic activity. However, homodimerization may be critical for the stability of the protein in the lysosomal environment. Supplementary Movie S1 provides detailed views into the hPLD3 active site, the N-glycosylation sites, the oxidized cysteine 300 and the homodimerization mode. The sequence comparison in the homodimerization region involving helices α10 and α11 shows that dimerization is also possible for hPLD4 (47% sequence identity and 77% similarity in 437 overlapping amino acids to hPLD3) (Supplementary Figure S2D, G), since the formation of the central hydrogen network is given by Arg363 and Asn360, whereas the stabilizing flanking hydrophobic contacts in hPLD3 Phe341 and Phe377 are replaced by Tyr354 and Tyr390 which provides potential to form hydrogen bonds (Supplementary Figure S2G), suggesting the possibility of forming a heterodimer.

hPLD3 revealed the highest 5′-exonuclease activity for a ssDNA oligomer starting at the 5′-end with a thymidine (Figure 3A). The phosphorothioate-modified ssDNA oligo T*TCATG in the active site of hPLD3 showed electron density only for the first 5′-thymidine and the phosphorothioate group of the second thymidine. That suggests that the remainder of the oligomer is either flexible or hydrolyzed. For our structural and biochemical studies, we used oligomers with phosphorothioate internucleotide linkages (*) either between the first two thymidines in T*T and T*TCATG or in all positions in T*T*C*A*T*G*, in whicha non-bridging oxygen is replaced by a sulfur atom. The oligomers were obtained as mixed chiral (Rp)- and (Sp)-configured enantiomers. The phosphorothioate linkages share similar physical and chemical properties with phosphodiesters but show enhanced nuclease tolerance compared to DNA/RNA. The unmodified TTCATG and the T*TCATG modified ssDNA bound to PLD3 in ITC experiment (Figure 4B, C), whereas the T*T and T*T*C*A*T*G* revealed no binding (Supplementary Figure S5A, B). We assume that T*T is too short to establish sufficient contacts for efficient binding, whereas the highly modified T*T*C*A*T*G* may not interact due to small differences in bond length and charge of the phosphorothioate backbone compared to a prochiral phosphate backbone. The titration of TTCATG to hPLD3 results in a negative ΔH and a positive -TΔS, which indicates an interaction that is dominated by hydrogen bond formation, while the interaction with T*TCATG with a positive ΔH and a negative -TΔS is mainly characterized by hydrophobic interactions. The hPLD3 crystal structure with the 3′-phosphorothioate thymidine of the ssDNA T*TCATG bound in the active site underlines the hydrophobic interaction by the stacking of the thymine base to Y411, which is stabilized by a few hydrogen bonds to the phosphorothioate. For the unmodified ssDNA TTCATG, a slightly different binding mode could be envisaged, which may be dominated by more hydrogen bonds to the unmodified phosphate backbone, besides the stacking interaction to Y411. Crystals for hPLD3 in complex with T*TCATG were grown in a crystallization condition containing sodium iodite. It has recently been reported that iodine preferentially attacks sulfur in phosphorothioate DNA and induces nucleolytic cleavage (48). Therefore, the bound 3′-phosphorothioate thymidine in the active site is most likely the cleavage product of T*TCATG, which further underlines the 5′-exonuclease function of hPLD3. Data from hPLD3 co-crystallized with the unmodified TTCATG revealed a 5′-thymidine in the active site, confirming this is the natural cleavage product. From the structural point of view, it is unclear why hPLD3 shows the lowest 5′-exonuclease activity for the 5′- cytidine end (5′-C), whereas an activity reduction for 5′-A and 5′-G oligomers can be explained by the loss of the hydrogen bond to the water molecule mediating the contact to Asp220.

PLD3 is a glycoprotein, and N-glycosylation is assumed to protect lysosomal enzymes from the harsh proteolytic conditions of the lysosomes with its high concentrations of proteases and peptidases. It is notable that the N-glycans of PLD3 are spread over the whole surface of hPLD3, even close to the dimerization interface, suggesting efficient protection of the dimer against proteolytic degradation (Figure 2F). Access to the catalytic site, however, is not hindered by the N-glycans.

Cysteine is the most reactive member of the natural proteinogenic amino acids and is a critical component in redox signaling. It has been shown that the oxidation of cysteine residues can alter the activities and function of several proteins in cells (49–51). Cysteine oxidation to cysteine sulfonic acid (OCS) is considered an irreversible posttranslational modification, often viewed as a hallmark of oxidative stress with extensive links to pathological neurodegeneration and myocardial diseases (52–55). The oxidative stress causes a profound change in the cardiomyocyte redox state (56). For the phospholipase D family member PLD2, an important signaling enzyme in mammalian cells, the oxidation state of its thiol groups by oxidants has a significant effect on the PLD activity and subsequent signal transduction process that influences cardiomyocyte function (54,57). The functional relevance of the OCS300 oxidation in hPLD3 is not clear, but it may act as a sensor of the hPLD3 redox state level in response to oxidative stress. The OCS300-connected manganese possibly contributes to the hyperoxidation of OCS300 due to the known ability of manganese to catalyze the oxidation of thiols (58). Also, hPLD4 has a proline-rich loop and a cysteine (Cys313) at the same position as the hyperoxidized cysteine 300 in hPLD3, making hyperoxidation possible (Supplementary Figure S2G).

Magnesium is by far the most frequently found metal ion cofactor in enzymatic systems like in endo- and exonucleases (59). Despite the high preference for Mg^2+^ supporting high levels of nucleolytic activity, a replacement by Mn^2+^ is comparable and effective. (60–62). Even distal metal ion binding sites of 20Å can have a drastic impact on the nucleolytic activity, as shown for the DNA restriction enzyme EcoRV (63). Also, in PLD3, the bound manganese and magnesium ions are located 20 Å distant from the active HKD/HKE site. The fact that the apo hPLD3 has an Mn^2+^ bound and the oligo complex an Mg^2+^ may be comparable to the reduction of star activity (relaxed specificity) as observed for some DNA restriction enzymes by the substitution of Mg^2+^ by Mn^2+^ (64). The bound manganese in the hPLD3 apo structure may provide hPLD3 with a higher exonuclease sequence specificity for substrate recognition, which is not necessary after substrate (oligo) binding and replaced by magnesium as seen in the hPLD3/oligo complex structure.

**Figure 5.**
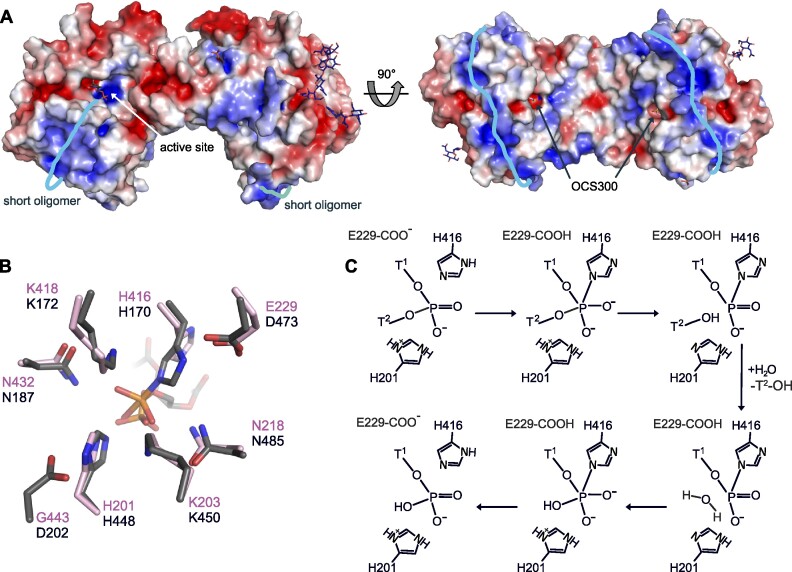
Model for ssDNA/RNA binding and hydrolysis. (**A**) Electrostatic surface potential of hPLD3 homodimer ranging from −3 kT/e (red) to +3 kT/e (blue) determined by the Pymol Plugin APBS Electrostatics tool. The potential oligomer binding route is depicted as light blue lines. (**B**) Superimposition of hPLD3 (pink) active-site residues with the structure of the *Streptomyces sp*. strain PMF phospholipase D (PDB code: 1V0Y) in dark gray. (**C**) Proposed 5′-exonuclease cleavage mechanism of hPLD3, which requires Glu229 as an acceptor (E229-COO^−^) in the upper scheme to accept the proton from His416 and release it again (E229-COOH) in the final step. His 201 is introduced in its protonated form so that it can protonate the cleaved T^2^-OH. In its resulting deprotonated form, it can activate the water molecule for nucleophilic attack on the phospho-histidine. At the end of the reaction cycle, all active site groups are back to their original state (His 416, E229-COO^−^, His 201), as required for an enzyme with turnover.

Investigating the electrostatic surface potential of the hPLD3 homodimer suggests where the ssDNA oligomers might bind before 5′-exonuclease cleavage (Figure 5A). Based on the phospholipid hydrolysis reaction model for the phospholipase D from *Streptomyces sp*. strain PMF (PDB Code = 1V0Y) and the highly similar sequence and structural arrangement for the active site residues with hPLD3 (Figure 5B), we postulate a model for the 5′-exonuclease cleavage process in hPLD3 (Figure 5C). In analogy to phospholipid hydrolysis (65), His416 may form a pentacovalent intermediates with the phosphate group of DNA. After cleavage and release of the second nucleotide thymidine (T2-OH), His201 may activate a water molecule for hydrolysis of the phospho-histidine intermediate, whereas the active-site lysines may help to compensate for excess negative charge developing during the transition state of the reaction.

Rare coding variants in *PLD3* were shown to double the risk for Alzheimer's disease and the overexpression of PLD3 reduced the levels of intracellular APP and extracellular Aβ42 and Aβ40 (12). However, this genetic link was challenged by several other groups later (13–16), and the effect of PLD3 on Aβ species was assigned to the strong overexpression in follow-up work (17). While further work is needed to clarify the potential role of *PLD3* as an Alzheimer's disease risk gene or disease modulator, additional work is also required to clarify the pathway(s) where it might modulate disease. However, our work shows that some of the mutations found in rare cases might well affect the function of the enzyme, including those mutations of the N-glycosylated residues N236 and N284. Intriguingly, the Cys300, which is oxidized to sulfonic acid, was also among the Alzheimer's disease-associated mutations. Further work will be required to determine the relevance of this cysteine for the function of PLD3.

*In vivo* the lysosomal turnover of nucleic acids mediated by PLD3 is critical for a balanced innate immune regulation via toll-like receptors (4,5). Consequently, fine-tuning PLD3’s activity by specific inhibitors or activators might both work as immunostimulatory and immunosuppressive therapeutic strategies and has the potential for the development of future immunotherapies, adjuvants, or nucleic acid drug stabilizers. Our high-resolution crystal structure will allow a targeted, rational, structure-based design of such modulators and help in the process of the development of nucleic-acid-turnover modulating drugs.

## Supplementary Material

gkad1114_Supplemental_Files

## Data Availability

Atomic coordinates of apo and DNA-bound hPLD3 have been deposited in the pdb database under accession codes 8Q1K and 8Q1X, respectively. All raw data have been uploaded to the ProteomeXchange Consortium (66) via the PRIDE partner repository with accession PXD044504. Raw data and graphs for the EFQO assays are available online (https://doi.org/10.5281/zenodo.10047708). All other primary data underlying this article will be shared on reasonable request to the corresponding author.

## References

[1] Fujiwara Y. , Wada K., Kabuta T. Lysosomal degradation of intracellular nucleic acids-multiple autophagic pathways. J. Biochem. 2017; 161:145–154. 28039390 10.1093/jb/mvw085

[2] Lardeux B.R. , Heydrick S.J., Mortimore G.E. RNA degradation in perfused rat liver as determined from the release of [14C]cytidine. J. Biol. Chem. 1987; 262:14507–14513. 2444586

[3] Blasius A.L. , Beutler B. Intracellular toll-like receptors. Immunity. 2010; 32:305–315. 20346772 10.1016/j.immuni.2010.03.012

[4] Gavin A.L. , Huang D., Blane T.R., Thinnes T.C., Murakami Y., Fukui R., Miyake K., Nemazee D. Cleavage of DNA and RNA by PLD3 and PLD4 limits autoinflammatory triggering by multiple sensors. Nat. Commun. 2021; 12:5874. 34620855 10.1038/s41467-021-26150-w PMC8497607

[5] Gavin A.L. , Huang D., Huber C., Martensson A., Tardif V., Skog P.D., Blane T.R., Thinnes T.C., Osborn K., Chong H.S. et al. PLD3 and PLD4 are single-stranded acid exonucleases that regulate endosomal nucleic-acid sensing. Nat. Immunol. 2018; 19:942–953. 30111894 10.1038/s41590-018-0179-y PMC6105523

[6] Evans C.J. , Aguilera R.J. DNase II: genes, enzymes and function. Gene. 2003; 322:1–15. 14644493 10.1016/j.gene.2003.08.022

[7] Kawane K. , Fukuyama H., Kondoh G., Takeda J., Ohsawa Y., Uchiyama Y., Nagata S. Requirement of DNase II for definitive erythropoiesis in the mouse fetal liver. Science. 2001; 292:1546–1549. 11375492 10.1126/science.292.5521.1546

[8] Bernardi A. , Bernardi G. Studies on acid hydrolases. IV. Isolation and characterization of spleen exonuclease. Biochim. Biophys. Acta. 1968; 155:360–370. 4295294

[9] Thorn A. , Steinfeld R., Ziegenbein M., Grapp M., Hsiao H.H., Urlaub H., Sheldrick G.M., Gartner J., Kratzner R. Structure and activity of the only human RNase T2. Nucleic Acids Res. 2012; 40:8733–8742. 22735700 10.1093/nar/gks614 PMC3458558

[10] Bernardi A. , Cantoni G.L. Action of spleen exonuclease on transfer ribonucleic acid. J. Biol. Chem. 1969; 244:1468–1476. 5773050

[11] Gonzalez A.C. , Schweizer M., Jagdmann S., Bernreuther C., Reinheckel T., Saftig P., Damme M. Unconventional trafficking of mammalian phospholipase D3 to lysosomes. Cell Rep. 2018; 22:1040–1053. 29386126 10.1016/j.celrep.2017.12.100

[12] Cruchaga C. , Karch C.M., Jin S.C., Benitez B.A., Cai Y., Guerreiro R., Harari O., Norton J., Budde J., Bertelsen S. et al. Rare coding variants in the phospholipase D3 gene confer risk for Alzheimer's disease. Nature. 2014; 505:550–554. 24336208 10.1038/nature12825 PMC4050701

[13] Hooli B.V. , Lill C.M., Mullin K., Qiao D., Lange C., Bertram L., Tanzi R.E. PLD3 gene variants and Alzheimer's disease. Nature. 2015; 520:E7–E8. 25832413 10.1038/nature14040

[14] Lambert J.C. , Grenier-Boley B., Bellenguez C., Pasquier F., Campion D., Dartigues J.F., Berr C., Tzourio C., Amouyel P. PLD3 and sporadic Alzheimer's disease risk. Nature. 2015; 520:E1. 25832408 10.1038/nature14036

[15] van der Lee S.J. , Holstege H., Wong T.H., Jakobsdottir J., Bis J.C., Chouraki V., van Rooij J.G., Grove M.L., Smith A.V., Amin N. et al. PLD3 variants in population studies. Nature. 2015; 520:E2–E3. 25832410 10.1038/nature14038 PMC4544703

[16] Heilmann S. , Drichel D., Clarimon J., Fernandez V., Lacour A., Wagner H., Thelen M., Hernandez I., Fortea J., Alegret M. et al. PLD3 in non-familial Alzheimer's disease. Nature. 2015; 520:E3–E5. 25832411 10.1038/nature14039

[17] Fazzari P. , Horre K., Arranz A.M., Frigerio C.S., Saito T., Saido T.C., De Strooper B. PLD3 gene and processing of APP. Nature. 2017; 541:E1–E2. 28128235 10.1038/nature21030

[18] Akizuki S. , Ishigaki K., Kochi Y., Law S.M., Matsuo K., Ohmura K., Suzuki A., Nakayama M., Iizuka Y., Koseki H. et al. PLD4 is a genetic determinant to systemic lupus erythematosus and involved in murine autoimmune phenotypes. Ann. Rheum. Dis. 2019; 78:509–518. 30679154 10.1136/annrheumdis-2018-214116

[19] Van Acker Z.P. , Perdok A., Hellemans R., North K., Vorsters I., Cappel C., Dehairs J., Swinnen J.V., Sannerud R., Bretou M. et al. Phospholipase D3 degrades mitochondrial DNA to regulate nucleotide signaling and APP metabolism. Nat. Commun. 2023; 14:2847. 37225734 10.1038/s41467-023-38501-w PMC10209153

[20] Varela-Ramirez A. , Abendroth J., Mejia A.A., Phan I.Q., Lorimer D.D., Edwards T.E., Aguilera R.J. Structure of acid deoxyribonuclease. Nucleic Acids Res. 2017; 45:6217–6227. 28369538 10.1093/nar/gkx222 PMC5449587

[21] Selvy P.E. , Lavieri R.R., Lindsley C.W., Brown H.A. Phospholipase D: enzymology, functionality, and chemical modulation. Chem. Rev. 2011; 111:6064–6119. 21936578 10.1021/cr200296t PMC3233269

[22] Pedersen K.M. , Finsen B., Celis J.E., Jensen N.A. Expression of a novel murine phospholipase D homolog coincides with late neuronal development in the forebrain. J. Biol. Chem. 1998; 273:31494–31504. 9813063 10.1074/jbc.273.47.31494

[23] Nishimasu H. , Ishizu H., Saito K., Fukuhara S., Kamatani M.K., Bonnefond L., Matsumoto N., Nishizawa T., Nakanaga K., Aoki J. et al. Structure and function of Zucchini endoribonuclease in piRNA biogenesis. Nature. 2012; 491:284–287. 23064230 10.1038/nature11509

[24] Ipsaro J.J. , Haase A.D., Knott S.R., Joshua-Tor L., Hannon G.J. The structural biochemistry of Zucchini implicates it as a nuclease in piRNA biogenesis. Nature. 2012; 491:279–283. 23064227 10.1038/nature11502 PMC3493678

[25] Stuckey J.A. , Dixon J.E. Crystal structure of a phospholipase D family member. Nat. Struct. Biol. 1999; 6:278–284. 10074947 10.1038/6716

[26] Ivics Z. , Hackett P.B., Plasterk R.H., Izsvak Z. Molecular reconstruction of Sleeping Beauty, a Tc1-like transposon from fish, and its transposition in human cells. Cell. 1997; 91:501–510. 9390559 10.1016/s0092-8674(00)80436-5

[27] Reeves P.J. , Callewaert N., Contreras R., Khorana H.G. Structure and function in rhodopsin: high-level expression of rhodopsin with restricted and homogeneous N-glycosylation by a tetracycline-inducible N-acetylglucosaminyltransferase I-negative HEK293S stable mammalian cell line. Proc. Natl. Acad. Sci. U.S.A. 2002; 99:13419–13424. 12370423 10.1073/pnas.212519299 PMC129688

[28] Markmann S. , Krambeck S., Hughes C.J., Mirzaian M., Aerts J.M., Saftig P., Schweizer M., Vissers J.P., Braulke T., Damme M. Quantitative proteome analysis of mouse liver lysosomes provides evidence for mannose 6-phosphate-independent targeting mechanisms of acid hydrolases in mucolipidosis II. Mol. Cell. Proteomics. 2017; 16:438–450. 28062798 10.1074/mcp.M116.063636 PMC5341004

[29] Cappel C. , Gonzalez A.C., Damme M. Quantification and characterization of the 5' exonuclease activity of the lysosomal nuclease PLD3 by a novel cell-based assay. J. Biol. Chem. 2021; 296:100152. 33288674 10.1074/jbc.RA120.015867 PMC7857491

[30] Krug M. , Weiss M.S., Heinemann U., Mueller U. XDSAPP: a graphical user interface for the convenient processing of diffraction data using XDS. J. Appl. Crystallogr. 2012; 45:568–572.

[31] Pape T. , Schneider T.R. HKL2MAP: a graphical user interface for macromolecular phasing with SHELX programs. J. Appl. Cryst. 2004; 37:843–844.

[32] Langer G. , Cohen S.X., Lamzin V.S., Perrakis A. Automated macromolecular model building for X-ray crystallography using ARP/wARP version 7. Nat. Protoc. 2008; 3:1171–1179. 18600222 10.1038/nprot.2008.91 PMC2582149

[33] McCoy A.J. , Grosse-Kunstleve R.W., Adams P.D., Winn M.D., Storoni L.C., Read R.J. Phaser crystallographic software. J. Appl. Crystallogr. 2007; 40:658–674. 19461840 10.1107/S0021889807021206 PMC2483472

[34] Casañal A. , Lohkamp B., Emsley P. Current developments in Coot for macromolecular model building of electron cryo-microscopy and crystallographic data. Protein Sci. 2020; 29:1069–1078. 31730249 10.1002/pro.3791 PMC7096722

[35] Agirre J. , Atanasova M., Bagdonas H., Ballard C.B., Baslé A., Beilsten-Edmands J., Borges R.J., Brown D.G., Burgos-Mármol J.J., Berrisford J.M. et al. The CCP4 suite: integrative software for macromolecular crystallography. Acta Crystallogr D. Struct. Biol. 2023; 79:449–461. 37259835 10.1107/S2059798323003595 PMC10233625

[36] Liebschner D. , Afonine P.V., Baker M.L., Bunkóczi G., Chen V.B., Croll T.I., Hintze B., Hung L.W., Jain S., McCoy A.J. et al. Macromolecular structure determination using X-rays, neutrons and electrons: recent developments in Phenix. Acta Crystallogr. D Struct. Biol. 2019; 75:861–877. 31588918 10.1107/S2059798319011471 PMC6778852

[37] Williams C.J. , Headd J.J., Moriarty N.W., Prisant M.G., Videau L.L., Deis L.N., Verma V., Keedy D.A., Hintze B.J., Chen V.B. et al. MolProbity: more and better reference data for improved all-atom structure validation. Protein Sci. 2018; 27:293–315. 29067766 10.1002/pro.3330 PMC5734394

[38] Krissinel E. , Henrick K. Inference of macromolecular assemblies from crystalline state. J. Mol. Biol. 2007; 372:774–797. 17681537 10.1016/j.jmb.2007.05.022

[39] Schuck P. Size-distribution analysis of macromolecules by sedimentation velocity ultracentrifugation and lamm equation modeling. Biophys. J. 2000; 78:1606–1619. 10692345 10.1016/S0006-3495(00)76713-0 PMC1300758

[40] Laue T. , Shah B., Ridgeway T., Pelletier S., Harding S., Rowe A., Horton J. 1992; Cambridge.

[41] Liu Y.D. , Goetze A.M., Bass R.B., Flynn G.C. N-terminal glutamate to pyroglutamate conversion in vivo for human IgG2 antibodies. J. Biol. Chem. 2011; 286:11211–11217. 21282104 10.1074/jbc.M110.185041 PMC3064176

[42] van den Bedem H. , Wilson M.A. Shining light on cysteine modification: connecting protein conformational dynamics to catalysis and regulation. J. Synchrotron Radiat. 2019; 26:958–966. 31274417 10.1107/S160057751900568X PMC6613112

[43] Thomas J.A. , Mallis R., Sies H. Cellular Implications of Redox Signaling. World Scientific Publ 141–174.

[44] Xu D. , Rovira I I, Finkel T. Oxidants painting the cysteine chapel: redox regulation of PTPs. Dev. Cell. 2002; 2:251–252. 11879627 10.1016/s1534-5807(02)00132-6

[45] Lambeth T.R. , Riggs D.L., Talbert L.E., Tang J., Coburn E., Kang A.S., Noll J., Augello C., Ford B.D., Julian R.R. Spontaneous isomerization of long-lived proteins provides a molecular mechanism for the lysosomal failure observed in Alzheimer's disease. ACS Cent. Sci. 2019; 5:1387–1395. 31482121 10.1021/acscentsci.9b00369 PMC6716341

[46] Metrick C.M. , Peterson E.A., Santoro J.C., Enyedy I.J., Murugan P., Chen T., Michelsen K., Cullivan M., Spilker K.A., Kumar P.R. et al. Human PLD structures enable drug design and characterization of isoenzyme selectivity. Nat. Chem. Biol. 2020; 16:391–399. 32042197 10.1038/s41589-019-0458-4

[47] Bowling F.Z. , Salazar C.M., Bell J.A., Huq T.S., Frohman M.A., Airola M.V. Crystal structure of human PLD1 provides insight into activation by PI(4,5)P(2) and RhoA. Nat. Chem. Biol. 2020; 16:400–407. 32198492 10.1038/s41589-020-0499-8 PMC7117805

[48] Huang Q. , Lee G.Y., Li J., Wang C., Zhao R.L., Deng Z., Houk K.N., Zhao Y.L. Origin of iodine preferential attack at sulfur in phosphorothioate and subsequent P-O or P-S bond dissociation. Proc. Natl. Acad. Sci. U.S.A. 2022; 119:e2119032119. 35439051 10.1073/pnas.2119032119 PMC9169930

[49] Marino S.M. , Gladyshev V.N. Analysis and functional prediction of reactive cysteine residues. J. Biol. Chem. 2012; 287:4419–4425. 22157013 10.1074/jbc.R111.275578 PMC3281665

[50] Reddie K.G. , Carroll K.S. Expanding the functional diversity of proteins through cysteine oxidation. Curr. Opin. Chem. Biol. 2008; 12:746–754. 18804173 10.1016/j.cbpa.2008.07.028

[51] Finkel T. Signal transduction by reactive oxygen species. J. Cell Biol. 2011; 194:7–15. 21746850 10.1083/jcb.201102095 PMC3135394

[52] Andersen J.K. Oxidative stress in neurodegeneration: cause or consequence?. Nat. Med. 2004; 10(Suppl.):S18–S25. 15298006 10.1038/nrn1434

[53] Klaunig J.E. , Kamendulis L.M. The role of oxidative stress in carcinogenesis. Annu. Rev. Pharmacol. Toxicol. 2004; 44:239–267. 14744246 10.1146/annurev.pharmtox.44.101802.121851

[54] Tappia P.S. , Dent M.R., Dhalla N.S. Oxidative stress and redox regulation of phospholipase D in myocardial disease. Free Radic Biol. Med. 2006; 41:349–361. 16843818 10.1016/j.freeradbiomed.2006.03.025

[55] Garrido Ruiz D. , Sandoval-Perez A., Rangarajan A.V., Gunderson E.L., Jacobson M.P Cysteine oxidation in proteins: structure, biophysics, and simulation. Biochemistry. 2022; 61:2165–2176. 36161872 10.1021/acs.biochem.2c00349 PMC9583617

[56] Ferrari R. , Guardigli G., Mele D., Percoco G.F., Ceconi C., Curello S. Oxidative stress during myocardial ischaemia and heart failure. Curr. Pharm. Des. 2004; 10:1699–1711. 15134567 10.2174/1381612043384718

[57] Dai J. , Meij J.T., Dhalla V., Panagia V. Involvement of thiol groups in the impairment of cardiac sarcoplasmic reticular phospholipase D activity by oxidants. J. Lipid Mediat. Cell Signal. 1995; 11:107–118. 7780680 10.1016/0929-7855(94)00031-7

[58] Peinado J. , Toribio F., Pérez-Bendito D. Study of the mechanism of the manganese-catalysed oxidation of a thiol-containing organic compound. Analyst. 1987; 112:771–774.

[59] Cowan J.A. Structural and catalytic chemistry of magnesium-dependent enzymes. Biometals. 2002; 15:225–235. 12206389 10.1023/a:1016022730880

[60] Vermote C.L. , Vipond I.B., Halford S.E. EcoRV restriction endonuclease: communication between DNA recognition and catalysis. Biochemistry. 1992; 31:6089–6097. 1627552 10.1021/bi00141a019

[61] Vipond I.B. , Baldwin G.S., Halford S.E. Divalent metal ions at the active sites of the EcoRV and EcoRI restriction endonucleases. Biochemistry. 1995; 34:697–704. 7819265 10.1021/bi00002a037

[62] El-Deiry W.S. , Downey K.M., So A.G. Molecular mechanisms of manganese mutagenesis. Proc. Natl. Acad. Sci. U.S.A. 1984; 81:7378–7382. 6095289 10.1073/pnas.81.23.7378 PMC392149

[63] Sam M.D. , Horton N.C., Nissan T.A., Perona J.J. Catalytic efficiency and sequence selectivity of a restriction endonuclease modulated by a distal manganese ion binding site. J. Mol. Biol. 2001; 306:851–861. 11243793 10.1006/jmbi.2000.4434

[64] Robinson C.R. , Sligar S.G. Molecular recognition mediated by bound water. A mechanism for star activity of the restriction endonuclease EcoRI. J. Mol. Biol. 1993; 234:302–306. 8230215 10.1006/jmbi.1993.1586

[65] DeYonker N.J. , Webster C.E. Phosphoryl transfers of the phospholipase D superfamily: a quantum mechanical theoretical study. J. Am. Chem. Soc. 2013; 135:13764–13774. 24007383 10.1021/ja4042753

[66] Vizcaino J.A. , Deutsch E.W., Wang R., Csordas A., Reisinger F., Rios D., Dianes J.A., Sun Z., Farrah T., Bandeira N. et al. ProteomeXchange provides globally coordinated proteomics data submission and dissemination. Nat. Biotechnol. 2014; 32:223–226. 24727771 10.1038/nbt.2839 PMC3986813

