# Supplementary material for: Structural analysis of PLD3 reveals insights into the mechanism of lysosomal 5′ exonuclease-mediated nucleic acid degradation
Source: Nucleic Acids Res. 2023 Nov 22;52(1):370–84. doi: 10.1093/nar/gkad1114 (PMC10783504; doi:10.1093/nar/gkad1114)
Supplement: gkad1114_Supplemental_Files [file gkad1114_supplemental_files.zip › SUPPLEMENTAL DATA.pdf]

SUPPLEMENTAL DATA

Structural analysis of PLD3 reveals insights into the mechanism of lysosomal 5' exonuclease-mediated nucleic acid degradation

Yvette Roske[1†], Cedric Cappel[2†], Nils Cremer[3] , Patrick Hoffmann[2], Tomas Koudelka[4], Andreas Tholey[4], Udo Heinemann[1,5], Oliver Daumke[1,5§], Markus Damme[2§]

[1]Structural Biology, Max Delbrück Center for Molecular Medicine (MDC), 13125 Berlin, Germany

[2]Biochemical Institute, Christian-Albrechts-University of Kiel, Kiel, Germany

[3]Leibniz-Institut für Molekulare Pharmakologie (FMP), Robert-Rössle-Straβe 10, 13125 Berlin, Germany

[4]Institute of Experimental Medicine, University of Kiel, 24188 Kiel, Germany.

[5]Institute for Chemistry and Biochemistry, Freie Universität Berlin, 14195 Berlin, Germany

[†]Joint first authors.

[§]Joint last authors: Oliver Daumke, oliver.daumke@mdc-berlin.de, Markus Damme, mdamme@biochem.uni-kiel.de

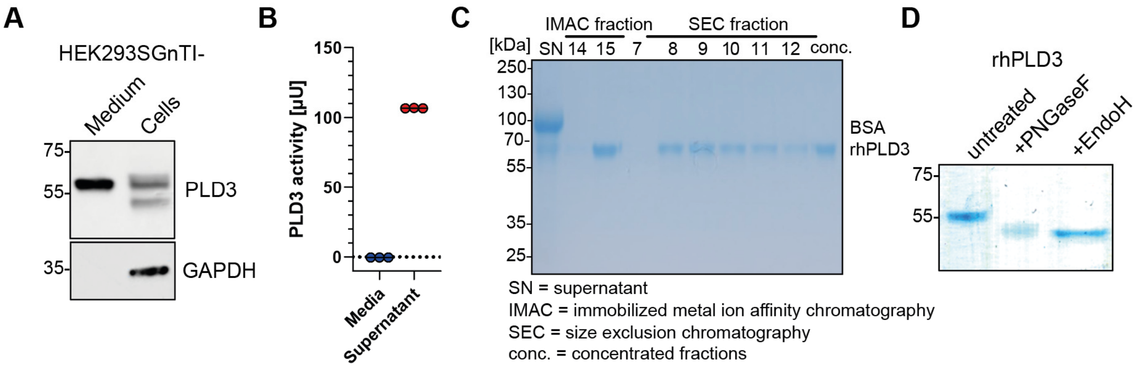

Supplementary Figure 1. Purification of hPLD3. (A) Immunoblot of conditioned cell culture medium (left) and lysates (right) from HEK293SGnTi- cells overexpressing hPLD3 with antibodies against PLD3 (luminal domain) and GAPDH. **(B)** EFQO activity assay of cell lysates and cell culture medium of HEK293SGnTi- cells overexpressing hPLD3. The cell culture medium was conditioned for seven days. **(C)** Coomassie-stained SDS-PAGE gel of fractions from different steps of the purification of the recombinant protein expressed in HEK293SGnTi- cells. **(D)** The purified recombinant hPLD3 was treated without or with peptide-N-glycosidase F (PNGaseF) or endoglycosidase H (EndoH) overnight. Both treatments effectively remove the N-glycans, indicating high-mannose-type N-glycosylation.

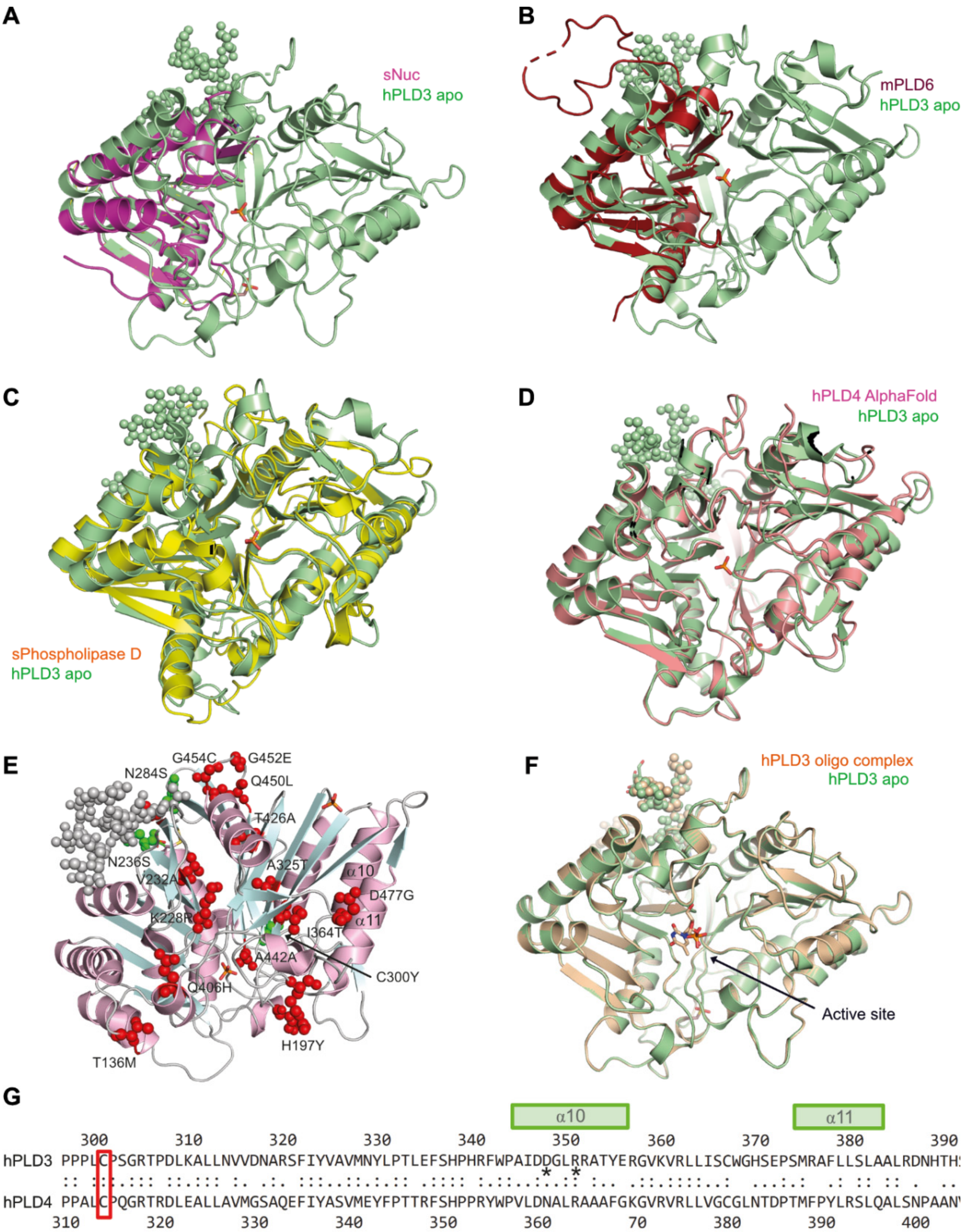

Supplementary Figure 2. Structural comparisons of hPLD3. Structural superimpositions of hPLD3 (green) onto **(A)** *Salmonella enterica* Nuc (PDB: 1BYR) in magenta with an rmsd of 1.076 Å, (B) mouse PLD6/zucchini (PDB: 4GGJ) in brown with rmsd = 1.320 Å, **(C)** *Seratia plymuthica* phospholipase D (PDB: 7e0m) in yellow with rmsd = 2.3 Å, **(D)** AlphaFold modeled hPLD4 in wheat with rmsd = 0.9249 Å. **(E)** hPLD3 residues mutated in Alzheimer's disease are depicted as red spheres. Mutations at the two glycosylated asparagines and the hyperoxidized cysteine 300 are highlighted as green spheres. **(F)** Superimposition of the apo-hPLD3 (green) and hPLD3- oligo complex form (wheat) revealed an rmsd of 0.114Å. **(G)** Cut-out of sequence alignment of hPLD3 and hPLD4 showing the similarity within the homodimerization area mediated by helices α10 and α11 (indicated with the green boxes above the sequence) and covering the proline-rich loop area in hPLD3 harboring the hyperoxidized Cys 300 (red box).

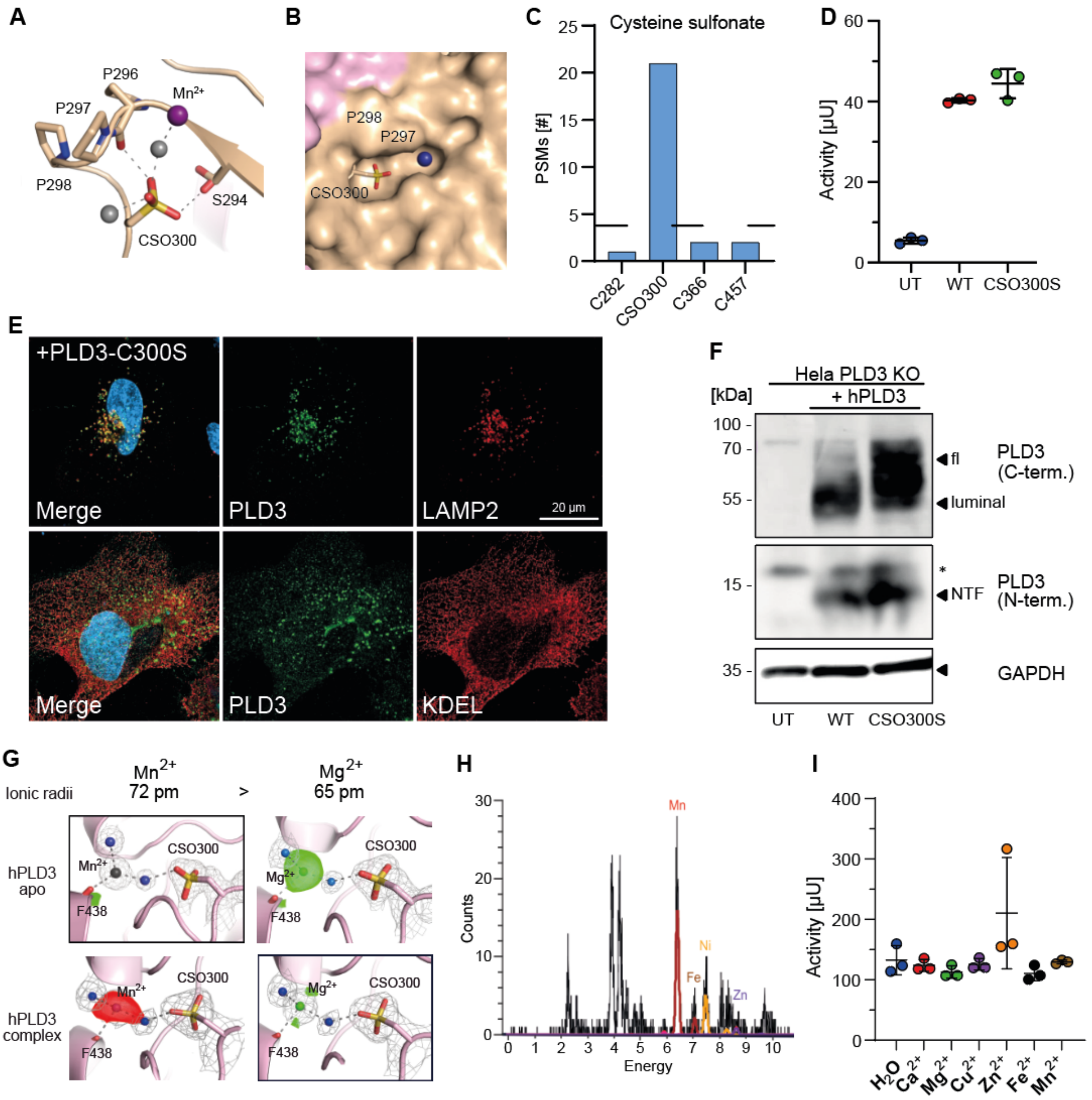

Supplementary Figure 3. The posttranslationally modified cysteine 300. (A) A magnified view of the posttranslationally modified cysteine sulfonic acid 300 (OCS300) of one protomer of the hPLD3 apo structure is shown as a stick representation coordinated by a proline-rich loop and an alternative conformation of Ser 294. The water-mediated bound manganese is depicted as a magenta sphere, and the water molecules as gray spheres. **(B)** Cut-out of the hPLD surface presentation to demonstrate the deep cavity harboring the OCS300 and the manganese ion in the hPLD3 apo structure (magnesium ion in hPLD3 oligo complex structure). **(C)** The number of peptide spectrum matches (PSMs) for cysteine sulfonate-modified cysteine by mass spectrometry. **(D)** EFQO PLD3 activity assay of lysates from untransfected Hela cells and cells transfected with wildtype PLD3 or with cysteine 300 mutated to serine. **(E)** Indirect immunofluorescence staining of Hela cells transfected with PLD3 with cysteine 300 mutated to serine with antibodies against PLD3 (green) and LAMP2 (red, upper panel) or KDEL (lower panel). Nuclei are stained with DAPI (blue). **(F)** Immunoblot of untransfected Hela cells and cells transfected with wildtype PLD3 or a mutant with cysteine 300 mutated to serine with antibodies against the C-terminus of PLD3 (upper panel) against the N-terminus (lower panel). GAPDH is depicted as a loading control. **(G)** Determination of divalent cation binding within the OCS300 cavity. The detailed view of protomer A is in the hPLD3 apo structure (upper two panels) and in the oligo bound complex structure (lower two panels) refined with placed Mn^{2+} in the left column and Mg^{2+} right column. Due to their different ionic radii, an Mn^{2+} in the hPLD3

apo structure and an Mg^{2+} in the hPLD oligo complex structure fit best, with no or nearly no difference in density. A positive difference density is shown at a +3 σ level as a green cloud and a negative density at -3 σ as a red cloud. The green difference density for Mg^{2+} in the apo structure (upper right) indicates that this cations's ionic radius is too small to fit well, whereas a Mn^{2+} at the same place in the hPLD3 complex structure is much too large for a proper fit, as indicated by the red difference density (lower left). **(H)** Fluorescence scan from an hPLD3 apo crystal reveals a prominent peak for manganese (red), smaller peaks for nickel (orange) and iron(brown), and a minimal peak for zinc (magenta). The absorption peaks for iron and nickel arise from the crystal mounting pin and can be therefore ignored. The very large manganese peak compared to the very small zinc peak clearly validates bound manganese in the hPLD3 apo structure. **(I)** 5'-Exonuclease activity of recombinant hPLD3 after removal of bound cations with EDTA and subsequent incubation with 100 µM magnesium or manganese (II), copper, zinc, iron, or calcium.

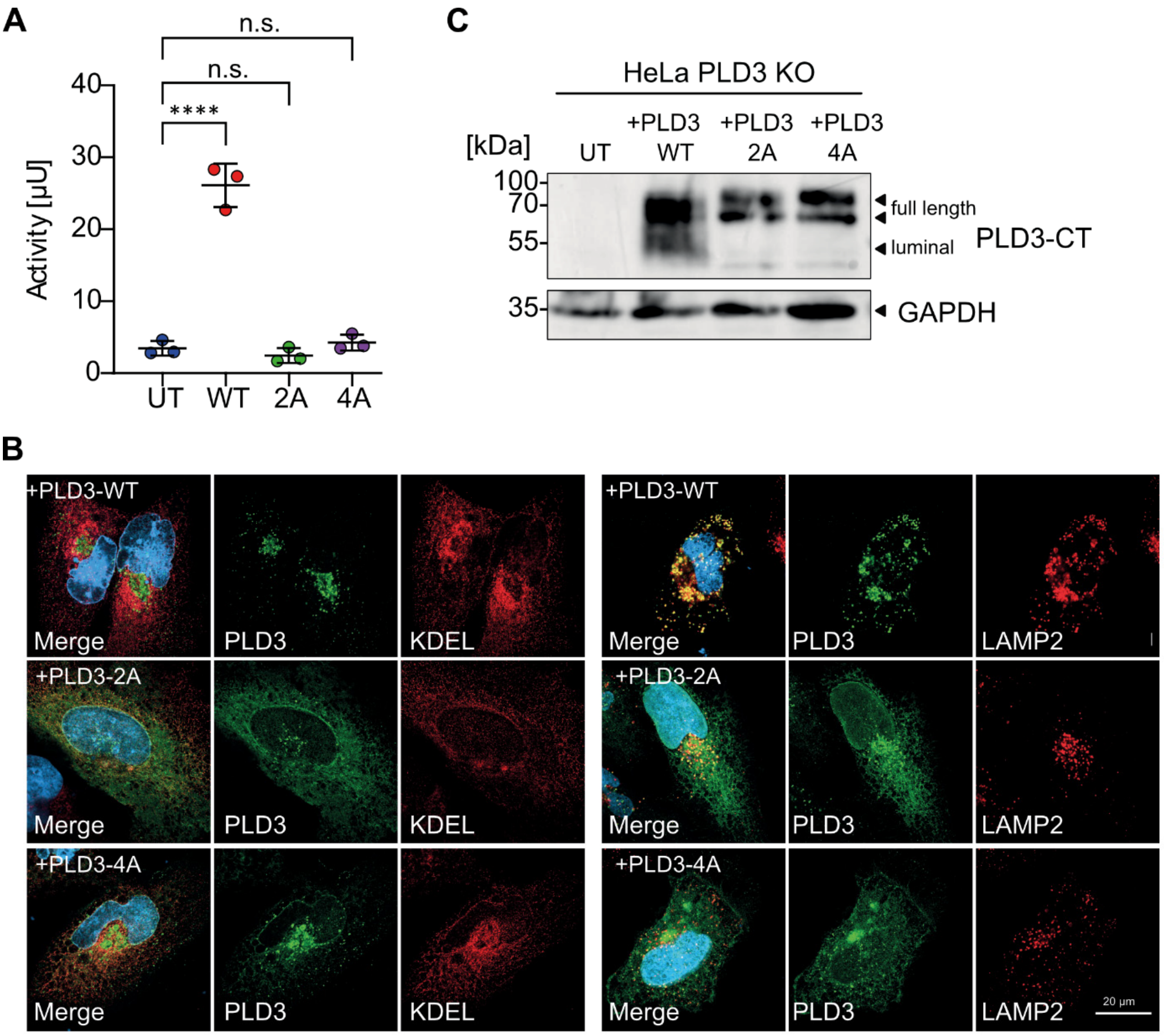

Supplementary Figure 4. Activity assay and indirect immunofluorescence staining of mutants in the dimerization interface. (A) Hela cells were transfected with cDNA corresponding to wildtype PLD3 or mutants in the dimerization interface (2A = R350A, S380A) and quadruple alanine mutants (4A = R350A, S380A, Y354A, F377A). The PLD3 activity was determined in cell lysates by the EFQO assay. **(B)** Indirect immunofluorescence staining of cells transfected with the dimerization interface-mutants stained with antibodies for PLD3 (green) and KDEL as a marker for the endoplasmic reticulum (left panel, red) or LAMP2 as a marker for lysosomes (right panel, red). **(C)** The proteolytic processing of the mutants was analyzed by immunoblot with an antibody against the luminal domain of PLD3.

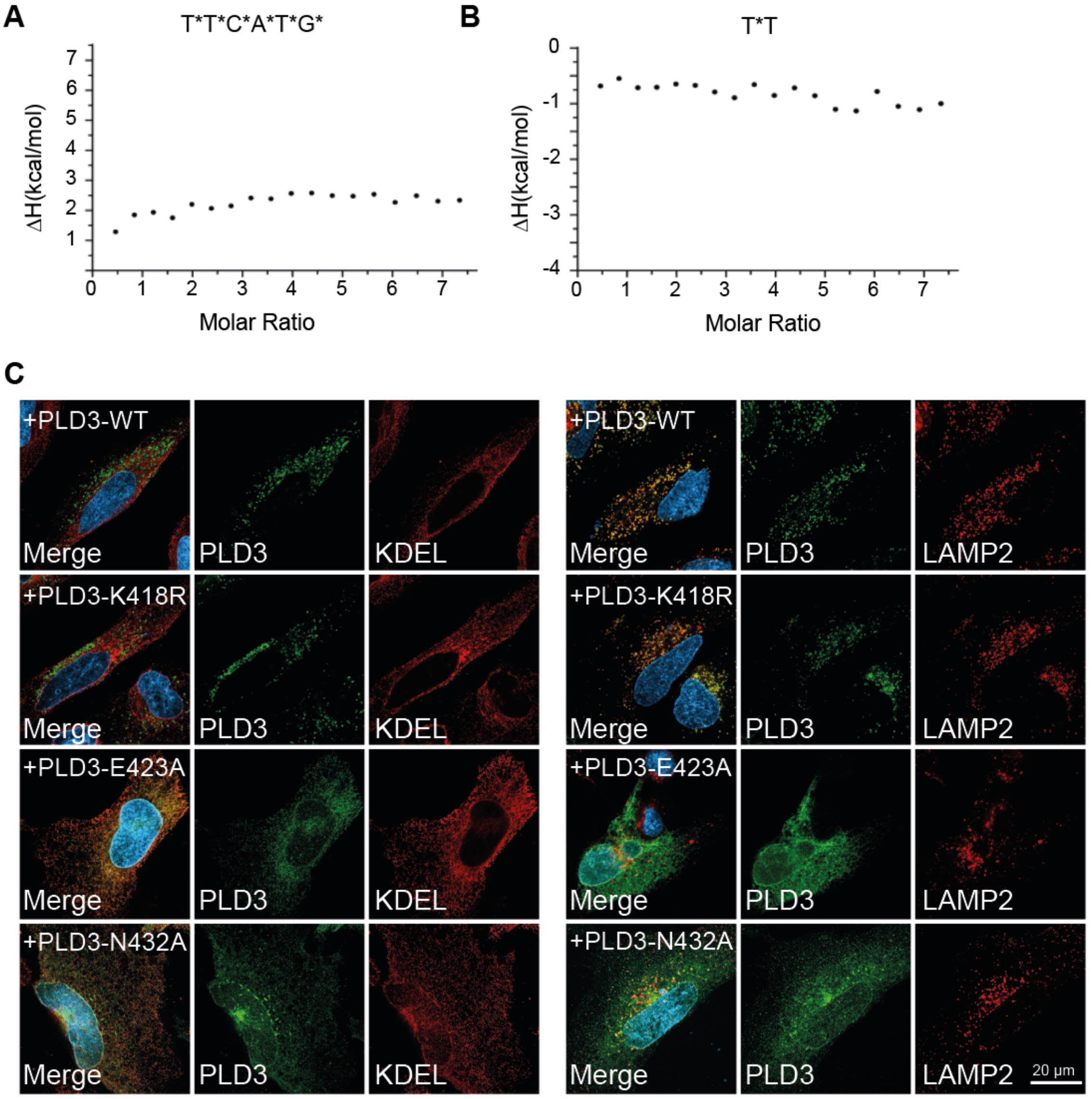

Supplementary Figure 5. Mutation of residues in the HKD1/HKE2 motif harboring the active site. ITC titration of ssDNA oligo variants to hPLD3 where the phosphorothioate modified T*T*C*A*T*G* **(A)** and T*T **(B)** oligo did not show any binding. **(C) Indirect immunofluorescence staining of active-site mutants.** Hela cells were transfected with cDNA corresponding to wildtype PLD3 (upper panel) or mutants in the active site (K418R, N432A) or a construct harboring a mutation in the HKE2 motif the overall structure stabilizing residue (E423A) and stained with antibodies for PLD3 (green) and KDEL as a marker for the endoplasmic reticulum (left panel, red) or LAMP2as a marker for lysosomes (right panel, red).

Supplementary Table 1

	hPLD3 (apo)	hPLD3 + T*TCATG
**Data collection**		
Space group	$P2_1$	$P2_1$
Cell dimensions		
a, b, c (Å)	60.2, 115.3, 101.1	60.5, 115.4, 101.3
α, β, γ (°)	90.0, 106.6, 90.0	90.0 106.6 90.0
Resolution (Å)*	44.6 - 1.51 (1.61 – 1.51)	48.6-1.85 (1.97-1.85)
R_{meas}*	7.4 (228.2)	14.7 (184.9)
< I / σ(I) >*	10.71 (0.64)	8.13 (0.82)
Completeness (%)*	97.8 (94.7)	98.4 (95.2)
Multiplicity	4.0	4.0
**Refinement**		
Resolution (Å)	1.51	1.85
No. unique reflections	202,454	111,277
R_{work} / R_{free} (%)	15.39/18.63	17.38/19.87
No. atoms		
Protein	6675	6671
Ligands	294	358
Water	1224	1036
Mean B factor ($Å^2$)	33.0	27.6
R.m.s deviations		
Bond lengths (Å)	0.005	0.009
Bond angles (º)	1.296	0.989
Ramachandran		
Outliers (%)	0.0	0.0
Allowed (%)	2.5	2.9
Favored (%)	97.5	97.1

*** Data in the highest resolution shell are indicated in parentheses.**
