# Supplementary material for: Structural analysis of PLD3 reveals insights into the mechanism of lysosomal 5′ exonuclease-mediated nucleic acid degradation
Source: Nucleic Acids Res. 2023 Nov 22;52(1):370–84. doi: 10.1093/nar/gkad1114 (PMC10783504; doi:10.1093/nar/gkad1114)
Supplement: gkad1114_Supplemental_Files [file gkad1114_supplemental_files.zip › SUPPLEMENTAL Video Legend.docx]

**SUPPLEMENTAL DATA**

**Structural analysis of PLD3 reveals insights into the mechanism of lysosomal 5' exonuclease-mediated nucleic acid degradation**

Yvette Roske^1†^, Cedric Cappel^2†^, Nils Cremer^3^ , Patrick Hoffmann^2^, Tomas Koudelka^4^, Andreas Tholey^4^, Udo Heinemann^1,5^, Oliver Daumke^1,5§^, Markus Damme^2§^

^1^Structural Biology, Max Delbrück Center for Molecular Medicine (MDC), 13125 Berlin, Germany

^2^Biochemical Institute, Christian-Albrechts-University of Kiel, Kiel, Germany

^3^Leibniz-Institut für Molekulare Pharmakologie (FMP), Robert-Rössle-Straβe 10, 13125 Berlin, Germany

^4^Institute of Experimental Medicine, University of Kiel, 24188 Kiel, Germany.

^5^Institute for Chemistry and Biochemistry, Freie Universität Berlin, 14195 Berlin, Germany

^†^Joint first authors.

^§^Joint last authors: Oliver Daumke, [oliver.daumke@mdc-berlin.de](mailto:oliver.daumke@mdc-berlin.de), Markus Damme, [mdamme@biochem.uni-kiel.de](mailto:mdamme@biochem.uni-kiel.de)

**Supplementary Video Legend:** Detailed views into the hPLD3 active site, the N-glycosylation sites, the oxidized cysteine 300, and the homodimerization mode
